# Machine learning intelligence to assess the shear capacity of corroded reinforced concrete beams

**DOI:** 10.1038/s41598-023-30037-9

**Published:** 2023-02-17

**Authors:** Aman Kumar, Harish Chandra Arora, Nishant Raj Kapoor, Krishna Kumar, Marijana Hadzima-Nyarko, Dorin Radu

**Affiliations:** 1 Academy of Scientific and Innovative Research (AcSIR), Ghaziabad, 201002 India; 2 Structural Engineering Department, CSIR-Central Building Research Institute Roorkee, Roorkee, 247667 India; 3 Department of Architecture and Planning, CSIR-Central Building Research Institute Roorkee, Roorkee, 247667 India; 4 Department of Hydro and Renewable Energy, Indian Institute of Technology Roorkee, Roorkee, 247667 India; 5 Faculty of Civil Engineering and Architecture Osijek, J. J. Strossmayer University of Osijek, Vladimira Preloga, Croatia; 6 Faculty of Civil Engineering, Transilvania University of Braşov, Braşov, Romania

**Keywords:** Civil engineering, Computational science

## Abstract

The ability of machine learning (ML) techniques to forecast the shear strength of corroded reinforced concrete beams (CRCBs) is examined in the present study. These ML techniques include artificial neural networks (ANN), adaptive-neuro fuzzy inference systems (ANFIS), decision tree (DT) and extreme gradient boosting (XGBoost). A thorough databank with 140 data points about the shear capacity of CRCBs with various degrees of corrosion was compiled after a review of the literature. The inputs parameters of the implemented models are the width of the beam, the effective depth of the beam, concrete compressive strength (CS), yield strength of reinforcement, percentage of longitudinal reinforcement, percentage of transversal reinforcement (stirrups), yield strength of stirrups, stirrups spacing, shear span-to-depth ratio (*a/d*), corrosion degree of main reinforcement, and corrosion degree of stirrups. The coefficient of determination of the ANN, ANFIS, DT, and XGBoost models are 0.9811, 0.9866, 0.9799, and 0.9998, respectively. The MAPE of the XGBoost model is 99.39%, 99.16%, and 99.28% lower than ANN, ANFIS, and DT models. According to the results of the sensitivity examination, the shear strength of the CRCBs is most affected by the depth of the beam, stirrups spacing, and the *a/d*. The graphical displays of the Taylor graph, violin plot, and multi-histogram plot additionally support the XGBoost model's dependability and precision. In addition, this model demonstrated good experimental data fit when compared to other analytical and ML models. Accurate prediction of shear strength using the XGBoost approach confirmed that this approach is capable of handling a wide range of data and can be used as a model to predict shear strength with higher accuracy. The effectiveness of the developed XGBoost model is higher than the existing models in terms of precision, economic considerations, and safety, as indicated by the comparative study.

## Introduction

One of the most important construction activities being carried out around the world today is the improvement of existing infrastructures. In terms of sustainable development, it is becoming necessary to upgrade old structures rather than demolish them. In many countries, design codes are constantly being updated due to increased load requirements that demand greater strength in structural components. Aging structures deteriorate over time as a result of environmental factors. Existing concrete structures can deteriorate due to reinforcement corrosion, carbonation, freeze–thaw cycles, etc. One of the most common causes of reinforced concrete (RC) elements degradation is corrosion, which reduces the cross-sectional area (A_cs_) of reinforcement bars, degrades their mechanical characteristics causes the concrete surface to crack or flake, and damages the steel to concrete bond^1^. Due to the gradual loss of the steel area, the propagation of the damage in the form of cracks and the final spalling of the concrete cover, and the deterioration of the connection between the steel reinforcement and the concrete, the bearing capacity of the corroding element decreases with the time of corrosion. Corrosion reduces the ability of the structures to support the load, which has an impact on both structural safety and in-service performance.

A well-known form of failure of RC elements is a shear failure which is very fast, without warning, failure, thus the RC elements must have sufficient shear capacity^2^. However, in service life, concrete structures undergo various types of environmental changes. Considering the corrosion, in the shear area, the small diameter of the stirrup and the brittle failure of the RC beam makes it more vulnerable. But still, these structure members take the load but when these damages increase more then it becomes very difficult to retain the loads. As shear failures are very dangerous, it is necessary to get early shear strength prediction using novel techniques.

A substantial amount of study has been conducted on many aspects of reinforcement corrosion relating to the corrosion process, its beginning, and undesirable impacts such as strength decrease and prediction of the residual strength of corroded components. The shear capacity of corroded RC beams (CRCBs) has recently been calculated using a variety of analytical models. Several formulas proposed by researchers, namely, Xu and Niu^3^, Yu^4^, Huo^5^, Zhao and Jin^6^, Li et al.^7^, Higgins et al.^8^, Webster^9^, Xue et al.^10^, and Khan et al.^11^ have been listed in the previous studies and used calculate the shear strength of the CRCBs. The shear performance of the CRCBs has been studied well through experimentation, however, extensive testing may be costly and time-consuming. The suggested models only take into account a small number of variables, leaving out certain significant ones that define the level of corrosion and other important parameters. As the analytical models were built on various model assumptions and various experimental databases, the accuracy and applicability of these models vary greatly under various situations. Additionally, the ability of analytical models to precisely forecast the shear capacity of degraded beams is constrained by the quick rise in corrosion levels in RC beams. Therefore, it is crucial to create methodologies that take into account all controlling factors. It is undoubtedly difficult to create an advanced shear prediction model that takes into account all the important factors since the shear capacity of CRCBs depends on a number of variables that are difficult to quantitatively characterize without making several assumptions.

Modern machine learning (ML) algorithms may offer a better solution to address these concerns because they are effective at handling complex problems involving many different factors without making any assumptions. These algorithms might be used to construct a shear capacity prediction model. Numerous services and sectors have seen significant productivity gains due to ML. Although it is still in its infancy in the construction sector, its application has grown recently to address several difficulties, including concrete technology^12–16^ and concrete durability^17–19^. The other various application in the field of civil and environmental engineering of ML algorithms can be found in the published research work^20–24^. The following is a brief description of some selected research related to the use of Extreme Gradient Boosting (XGBoost) and other ML (Classification and regression tree (CART), adaptive boosting (AdaBoost), gradient boosted decision trees (GBDT), support vector regression (SVR), random forest (RF), extremely random trees (ERT), artificial neural network (ANN), gene expression programming (GEP), Kernel ridge regression (KNN), K-nearest neighbor (KRR), Gaussian process regression (GPR) multivariate adaptive regression splines (MARS), support vector machine (SVM), linear regression (LR), decision tree (DT) ensemble tree (ET), and evolutionary polynomial regression (EPR)) algorithms in concrete technology and RC beams:

Wakjira et al.^25^ investigated the flexural capacity of FRP-reinforced RC beams. Six input parameters were used to develop the ML models. Five ML algorithms (CART, AdaBoost, GBDT, XGBoost, and Super-learner) were used to develop the most appropriate predictive model. The super-learner algorithm provides the highest predictive performance among all the models studied, with the lowest RMSE and MAPE and as the highest R^2^. In another study, the shear capacity of the shear-critical RC beams reinforced with novel composites was investigated by Wakjira et al. using ML techniques^26^. The prediction models were built using SVR, CART, RF, extremely random trees, GBDT, and XGBoost algorithms. According to the results of the study findings, the XGBoost model performs well compared to other ML techniques and existing guidelines. Uddin et al.^27^ used ANN, RF, GEP, and GBDT to predict the shear strength of RC beams. The performance of GBDT algorithm was good compared to ANN, RF and GEP algorithms. In another study, Wakjira et al.^28^ investigated the flexural capacity of FRCM-reinforced RC beams using KNN, KRR, SVR, CART, RF, GBDT and XGBoost methods. The XGBoost model shows good performance and having the highest R^2^-value of 99.3%, the lowest MAE, and the MAPE. The proposed model has better predictive power and robustness as shown by its performance with that of existing analytical models. Based on the above mentioned research work, the performance of the XGBoost algorithm was higher compared to other ML algorithms.

Badra et al.^29^ predicted the punching shear strength of FRP-reinforced concrete slabs without shear reinforcement using ANN and SVM algorithms. The RMSE value of the ACI, CSA, JSCE, ANN, and SVM models are 3.06 kN, 1.70 kN, 1.99 kN, 1.10 kN, and 1.32 kN, respectively. When comparing the performance metrics the performance of ANN was superior. Deifalla and Salem^30^ investigated the torsional strength of externally bonded FRP-reinforced concrete beams using ET, GPR and ANN models. The broad neural network model was the most effective model for predicting the torsion strength of RC beams strengthened with EB-FRP. The R^2^, RMSE, and MAE values of the models were 0.93, 16,634 kN and 0.98 kN, respectively, and they reported the best performance; however, they required the most training time. Mohammed and Ismail^31^ used MARS, XGBoost and SVM models to predict the shear strength of RC beams. According to the research results, the developed MARS and XGBoost models for simulating the shear strength of RC beams have potential. The results showed that all the beam geometry and concrete properties criteria used were important for building the prediction model. Numerically, the MARS model achieved the lowest possible RMSE (89.96 kN). Salem and Deifalla^32^ evaluated the strength of slab-column connections with FRPs using ML algorithms (LR, DT, SVM, ET, and GPR). The ideal hyper-parameters of the ML-based algorithms were selected during the training process using a grid search with a 15-fold cross-validation. Among all the applied ML models, the ensemble boosted model was found to be the most trustworthy and accurate model, with the best accuracy: R^2^, RMSE, and MAE were 0.97, 71.963 kN, and 43.452 kN, for the test dataset, respectively. Ebid and Deifalla^33^ used ML procedures to predict the punching shear capacity of lightweight concrete slabs. The column dimensions, concrete density, slab effective depth, CS, yield strength of steel, and flexural reinforcement ratio were considered as input parameters. The highest prediction accuracy is shared by ANN and EPR (73.9% and 73.6%, respectively), while the GP model has the lowest prediction accuracy (67.6%). Kaveh et al.^34^ used an XGBoost framework to calculate the shear capacity of FRP-strengthened concrete beams. The correlation coefficient of the developed XGBoost model was 0.94, which was higher than all the empirical models.

The aim of this article is to estimate the shear strength of CRCBs using ANN, ANFIS, DT, and XGBoost algorithms. To the best knowledge of the authors, DT, ANFIS, and XGBoost algorithms have not been previously used to estimate the shear strength of CRCBs. The influence of individual input parameters on the predicted shear capacity of CRCBs is also determined.

## Analytical models to estimate the shear capacity of CRCBs

### Xu and Niu’s model

The limit equilibrium theory serves as the foundation for the formula used by Xu and Niu^3^ to determine the shear strength of CRCBs. The impact of reinforcing steel corrosion on shear capacity is taken into consideration by adding a shear span-to-depth ratio (*a/d*) as well as the reduction in A_cs_ of the stirrup and yield strength due to corrosion. The formulation is expressed in Eqs. (1–4):

1 $${V}_{u}= {V}_{c}+{V}_{s}=\xi \left(\lambda ,{\eta }_{w,sn}\right)\times \left[\frac{\left(0.08+4{\rho }_{l}\right)}{\left(\lambda -0.3\right)}\right]\times {f}_{ck}b{h}_{o}+\alpha \left({\eta }_{w,sn}\right)\times \left(0.25+0.4\lambda \right){A}_{vc}{f}_{yv}\frac{{h}_{o}}{s}$$

2 $$\xi \left(\lambda ,{\eta }_{w,sn}\right)=\left\{\begin{array}{l}1, {\eta }_{w,sn}\le {\eta }_{cr,sn} \\ {\left(\frac{{\eta }_{w,sn}}{{\eta }_{cr,sn}}\right)}^{0.069\lambda -0.43}, {\eta }_{w,sn}>{\eta }_{cr,sn}\end{array}\right.$$

3 $${\eta }_{cr,sn}=10.4\frac{{c}_{v}}{{\phi }_{v}^{2}}+\frac{{f}_{cu,150}}{{\phi }_{v}}$$

4 $$\alpha \left({\eta }_{w,sn}\right)=1-1.077{\eta }_{w,sn}$$

where, ${V}_{u}$, ${V}_{c}$, and ${V}_{s}$ represents the shear resistance of the RC beam, concrete resistance, and stirrups shear resistance, respectively. $\xi \left(\lambda ,{\eta }_{w,sn}\right)$ and $\alpha \left({\eta }_{w,sn}\right)$ considered as reduction factors. $\lambda$, ${f}_{ck}$, ${f}_{yv}$ ${A}_{vc}$, ${f}_{cu,150}$, and ${\phi }_{v}$ are *a/d*, CS of concrete, stirrups yield strength, residual area of the stirrups, CS of concrete (150 mm cube), and diameter of stirrups, respectively. ${\rho }_{l}$, ${\eta }_{w,sn}$, and ${\eta }_{cr,sn}$ are the percentage of longitudinal steel, percentage of stirrups corrosion, and crack initiation of stirrups corrosion ratio, respectively. *b, *${h}_{o}$*,* $s$ and ${c}_{v}$ are the beam width, effective depth, stirrups spacing, and concrete cover of stirrups, respectively.

### Yu’s model

Yu has suggested a small change to the GB50010-2002^35^ standard guideline. A new coefficient was proposed by Yu^4^ to identify the impact of corrosion on longitudinal steel. The model also takes into account how corrosion affects the A_cs_ and stirrups yield strength^4^. The formulation to forecast the shear capacity of CRCBs is expressed in Eqs. (5–7):

5 $${V}_{u}=\frac{1.75\varphi {f}_{t}b{h}_{o}}{\left(\lambda +1\right)}+\frac{1.25{f}_{yc}{A}_{vc}{h}_{o}}{s}$$

6 $$\varphi = -0.0354{\eta }_{l,sn}^{2}+0.6256{n}_{l,sn}-1.2349$$

7 $${f}_{yc}=\frac{\left(0.985-1.028{\eta }_{w,sn}\right){f}_{yv}}{\left(1-{\eta }_{w,sn}\right)}$$

where, $\varphi$, ${f}_{yc}$, ${f}_{t}$ and ${n}_{l,sn}$ are the reduction factor, yield strength of stirrups (corroded), the tensile strength of concrete, and section loss ratio of main steel.

### Huo’s model

Huo^5^ introduced two reduction factors on the basis of the shear strength analytical model of an un-corroded RC beam proposed by the China Academy of Building Research in 1985^36^ to take into account stirrup corrosion and longitudinal steel of CRCBs. In experimental tests of CRCBs, regression analysis is used to determine both of these reduction variables^5^. The formulation to estimate the shear strength of CRCBs is given in Eqs. (8–10):

8 $${V}_{u}=\varphi {f}_{ck}b{h}_{o}\left[\frac{0.08}{\left(\lambda -0.3\right)}+\frac{100{\rho }_{l}}{\left(\lambda . {f}_{ck}\right)}\right]+\frac{\alpha \left(0.4+0.3\lambda \right){A}_{v}{f}_{yv}{h}_{o}}{s}$$

9 $$\varphi =\left\{\begin{array}{l}1.0, {\eta }_{l,wt}\le 5\% \\ 1.098-1.96{\eta }_{l,wt}, { \eta }_{l,wt}>5\%\end{array}\right.$$

10 $$\alpha =1-1.059{\eta }_{w.wt}$$

where, both $\varphi$ and $\alpha$ are the reduction factors (${\eta }_{l,wt}$ and ${\eta }_{w.wt}$), and ${A}_{v}$ is the un-corroded area of the stirrup.

### Zhao and Jin’s model

A methodology for estimating the shear capacity of un-corroded RC beams under two-point loading was provided by Zararis^37^. Zhao and Jin^6^ suggested a variation of Zararis's model, which is applied to calculate the shear strength of CRCBs. Zhao and Jin^6^ take into account a reduction factor that includes all of the effects of corrosion on stirrups. The formulation to forecast the shear capacity of CRCBs is expressed in Eqs. (11–13):

11 $${V}_{u}= \alpha {V}_{u0}= \alpha b{h}_{o}\left[\frac{{C}_{s}{f}_{cyl,150}\left(1-\frac{0.5{C}_{s}}{{h}_{o}}\right)}{{h}_{o}}+\frac{0.5{\rho }_{v}{f}_{yv}{\left(1-\frac{{C}_{s}}{{h}_{o}}\right)}^{2}{\left(\frac{a}{{h}_{o}}\right)}^{2}}{\left(\frac{a}{{h}_{o}}\right)}\right]$$

12 $$\alpha =\left\{\begin{array}{l}1.0, {\eta }_{w,sn}\le 10\%\\ 1.17-1.7{\eta }_{w,sn}, {\eta }_{w,sn}>10\%\end{array}\right.$$

13 $$\frac{{C}_{s}}{{h}_{o}}=\frac{1+0.27\left[{ \left(\frac{a}{{h}_{o}}\right)}^{2}+\frac{{\rho }_{v}}{{\rho }_{l}}\right]}{2\left[1+{\left(\frac{a}{{h}_{o}}\right)}^{2}+\frac{{\rho }_{v}}{{\rho }_{l}}\right]}\left[\sqrt{{\left(\frac{600{\rho }_{l}}{{f}_{cyl,150}}\right)}^{2}+4\left(\frac{600{\rho }_{l}}{{f}_{cyl,150}}\right)}-\frac{600{\rho }_{l}}{{f}_{cyl,150}}\right]$$

where, ${V}_{u0}$, $\alpha$, ${C}_{s}$, ${\rho }_{v}$, and ${f}_{cyl,150}$ are the ultimate shear resistance of uncorroded beams, shear span, compression zone depth, percentage steel of stirrups, and CS of concrete (150 × 300 mm specimens), respectively.

### Li et al.’s model

Li et al.^7^ also proposed an equation based on the Chinese Guideline (GB50010-2002)^35^ for the shear capacity estimation of CRCBs. The equation takes into account the change in height and width of the corrosion-damaged stirrups cross-section. Additionally, stirrups' corrosion and yielding strength are taken into consideration. The formulation to evaluate the shear capacity of CRCBs is expressed in Eqs. (14–17):

14 $${V}_{u}=\frac{1.75{f}_{t}{b}_{c}{h}_{oc}}{\left(\lambda +1\right)}+\frac{{f}_{yc}{A}_{vc}{h}_{oc}}{s}$$

15 $${f}_{yc}=\frac{\left(1-1.1219{\eta }_{w,wt}\right){f}_{yv}}{\left(1-{\eta }_{w,wt}\right)}$$

16 $${b}_{c}=b-{C}_{v1}-{C}_{v2}$$

17 $${h}_{0c}={h}_{0}-{C}_{sc}$$

where, ${C}_{v1}$ and ${C}_{v2}$ are concrete cover on both cross-sectional width directions.

### Lu et al.’s model

Lu et al.,^38^ incorporate the impacts of stirrups, the level of corrosion of longitudinal reinforcement, and the *a/d* in the shear strength estimation of the CRCBs. Overestimating the residual shear strength of CRCBs with stirrups and longitudinal reinforcement corrosion would be risky. In addition, diagonal tension failure and shear compression failure were also considered, and the maximum value is taken for final calculations. The shear strength of CRCBs under purposive stress is shown in Eqs. (18–23):

18 $${V}_{u}=\phi {V}_{c}+{V}_{s}$$

where, $\phi$ is a reduction coefficient linked with *a/d*.

Shear resistance of concrete (${V}_{c}$) can be calculated using Eq. (19)^39^:

19 $${V}_{c}=Max\left({V}_{c1},{V}_{c2}\right)$$

where, the terms ${V}_{c1}$ is shear resistance of concrete at diagonal tension and expressed in Eq. (20)^40^:

20 $${V}_{c1}=0.2\sqrt[3]{100{f}_{cyl,150}{\rho }_{lc}} \sqrt[4]{\frac{{10}^{3}}{{h}_{0}}}\left(0.75+\frac{1.4{h}_{0}}{a}\right)b{h}_{0}$$

where, ${\rho }_{lc}$ is the percentage of corroded longitudinal steel, and ${V}_{c2}$ represent the compression failure and can be determined by Eq. (21)^41^:

21 $${V}_{c2}=\frac{0.24\sqrt[3]{{f}_{cyl,150}^{2}}\left(1+\sqrt{100{\rho }_{lc}}\right)\left(1+\frac{3.33r}{{h}_{0}}\right)b{h}_{0}}{\left[1+{\left(\frac{a}{{h}_{0}}\right)}^{2}\right]}$$

where, $r$ is the width of the loading plate (87.2 mm).

Shear resistance of the stirrups (${V}_{s}$) is expressed in Eq. (22)^10^.

22 $${V}_{s}=\frac{{f}_{yv}{A}_{vc}j{h}_{0}}{s}$$

where, $j$ is a coefficient and is generally taken as 1/1.15^10^.

23 $$\phi =\left\{\begin{array}{c}0.008{e}^{\left(-0.122\lambda \right)}-0.003{\eta }_{w,sn}+1.01, \lambda <2.5\\ 0.1{e}^{\left(-0.122\lambda \right)}-0.003{\eta }_{w,sn}+1.38, \lambda \ge 2.5\end{array}\right.$$

## Machine learning models establishment

### Database setting up

**Figure 1:**
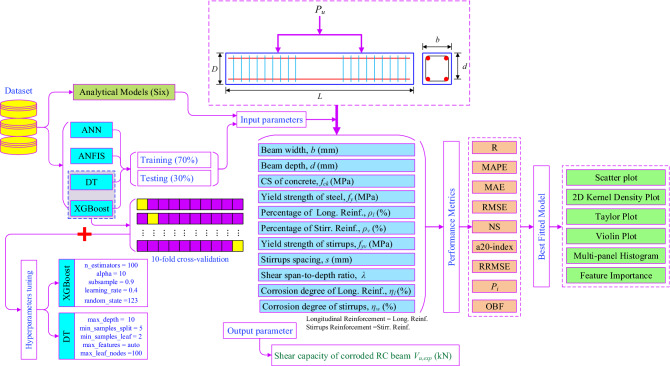
Methodology chart.

**Figure 2:**
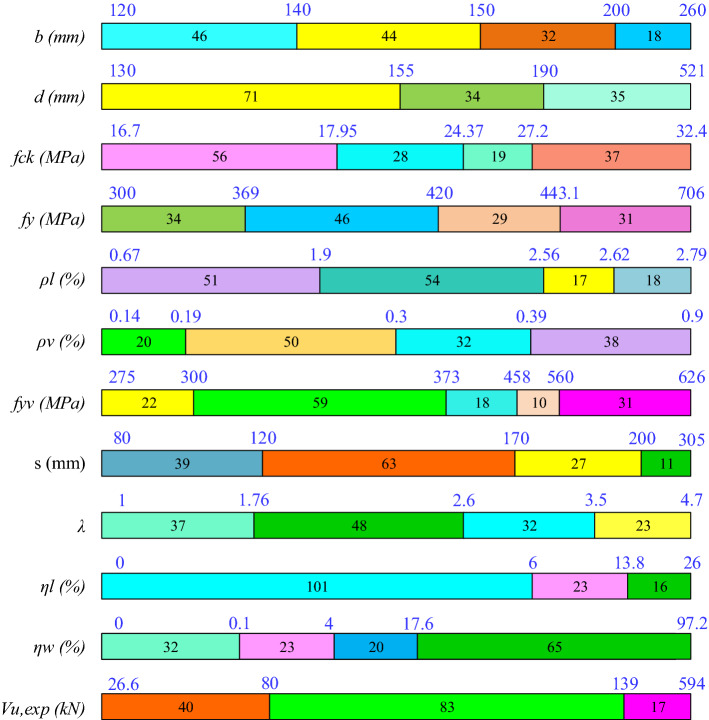
Summary of statistical analysis of the CRCBs.

A literature survey was conducted to collect experimental data on the shear capacity of CRCBs. 140 datasets have been collected from the reviewed literature^6,10,42–47^. The parameters that affect the shear strength of CRCBs are: (i) width of the beam (*b*), (ii) effective depth of the beam (*d*), (iii) CS of concrete (*f*_*ck*_), (iv) yield strength of reinforcement (*f*_*y*_), (v) percentage of longitudinal reinforcement (${\rho }_{l})$, (vi) percentage of stirrups reinforcement (${\rho }_{v})$, (vii) yield strength of stirrups (*f*_*yv*_), (viii) stirrups spacing (*s*), (ix) a/d, (x) corrosion degree of longitudinal reinforcement ((${\eta }_{l}$)), and (xi) corrosion degree of stirrups (${\eta }_{w}$). The same parameters have been used to develop ML models. The complete methodology to accomplish the objective of this work is depicted in Fig. 1 and explained in subsequent sections. Table 1 lists the statistical properties of the amassed database, and Fig. 2 displays the distribution of the collected parameters.

**Table 1:** Statistical parameters.

Index	*b* (mm)	*d* (mm)	*fck* (MPa)	*fy* (MPa)	*ρl* (%)	*ρv* (%)	*fyv* (MPa)	*s* (mm)	*λ*	*ƞl* (%)	*ƞw* (%)	*Vu,exp* (kN)
Mean	166.50	195.79	22.60	444.70	2.06	0.36	403.53	154.18	2.54	3.89	21.85	116.60
Std	45.60	92.84	5.59	96.22	0.62	0.19	105.06	46.89	0.98	6.25	25.20	89.47
Min	120	130	16.7	300	0.67	0.14	275	80	1	0	0	26.60
25%	120	150	17.33	390	1.65	0.23	332	120	1.76	0	0.19	80
50%	150	155	21.09	417.80	2.15	0.31	369.60	150	2.20	0	15.50	96.05
75%	200	207.50	27.89	443.10	2.58	0.45	467	200	3.10	8.73	32.60	122.40
Max	260	521	32.4	706	2.79	0.90	626	305	4.70	26	97.20	594
Skewness	0.83	1.93	0.36	1.26	− 0.86	1.53	0.73	0.40	0.66	1.35	1.31	3.66
Kurtosis	2.54	6.62	1.58	3.99	2.94	4.72	2.36	3.03	2.51	3.62	4.12	1.78

**Figure 3:**
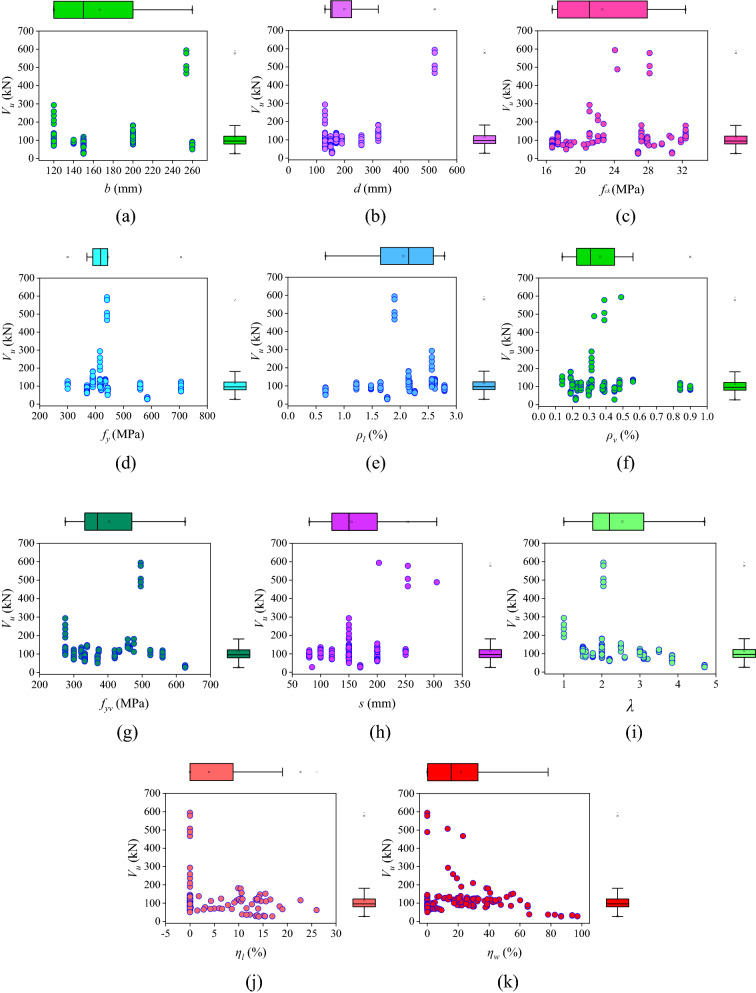
Marginal box chart of input parameters with shear capacity of CRCBs (**a**) *b*, (**b**) *d*, (**c**) *f*_*ck*_, (**d**) *f*_*y*_, (**e**) ${\rho }_{l}$, (**f**) ${\rho }_{v}$, (**g**) *f*_*yv*_, (**h**) *s*, (**i**) $\lambda$, (**j**) ${\eta }_{l}$ and (**k**) ${\eta }_{w}$.

To determine the relationship between input parameters and shear capacity, and to show the dot distribution a marginal plot is used, as shown in Fig. 3. A scatterplot with histograms, boxplots, or dot-plots in the x and y-axes' margins is referred to as a marginal plot. Figure 3a–k shows the marginal plot of all the input parameters like *b*, *d*, *f*_*ck*_, *f*_*y*_, ${\rho }_{l}$, ${\rho }_{v}$, *f*_*yv*_, *s*, $\lambda$, ${\eta }_{l}$ and ${\eta }_{w}$, respectively.

#### Data preparation

In all ML algorithms, it is necessary to standardize the dataset in a certain form. The process of standardization increases the efficiency and accuracy of ML algorithms. The commonly used standardization ranges are: (i) 0–1, (ii) − 1 to + 1, and (iii) 0–9. In this study, the − 1 to + 1 standardized range has been adopted to normalize the collected parameters. The formulation used for normalization is expressed in Eq. (24)^48^:

24 $${Z}_{normalized}=\left[2\times \frac{\left(z-{z}_{min}\right)}{\left({z}_{max}- {z}_{min}\right)}\right]-1$$

where, ${Z}_{normalized}$ is the normalized outcome, *z* is the value to be standardized in the selected dataset, ${z}_{min}$ and ${z}_{max}$ are the minimum and maximum values in the selected dataset, sequentially.

After normalization, the dataset has been processed for further processing in the different phases such as the training, and testing phases. In ANN, ANFIS, DT, and XGBoost models, the dataset has been categorized only in the training and testing phases with a percentage of 70% and 30%, respectively (Fig. 1).

#### Model evaluation

To estimate the performance of analytical and ML models, the used performance metrics are: correlation coefficient (R), root mean square error (RMSE), Nash–Sutcliffe efficiency index (NSEI), mean absolute percentage error (MAPE), and mean absolute error (MAE). In addition, the performance index (*P*_*i*_) and over-fitting analysis (OFA) have also been done to check the fitting of the ML algorithms. The formulation of all the performance metrics is given in Table 2^49,50^.

**Table 2:** Description of performance metrics.

S. no.	Performance Metric	Formulation	Description
1	R	$\frac{{\sum }_{i=1}^{N}\left({E}_{i}-\overline{E }\right)\left({P}_{i}-\overline{P }\right)}{\sqrt{{\sum }_{i=1}^{N}{\left({E}_{i}-\overline{E }\right)}^{2}{\sum }_{i=1}^{N}{\left({P}_{i}-\overline{P }\right)}^{2}}}$	Where,* E* and* P* are the experimental and predicted output sets respectively, $\overline{E }$ and $\overline{P }$ are the mean of experimental and predicted output sets respectively, *N* is the number of points in the dataset, and *m20* is the number of values obtained from measured/predicted value and lies in the range of 0.8–1.2
2	MAPE	$\frac{1}{N}\sum_{i=1}^{N}\left|{E}_{i}-{P}_{i}\right|$	
3	RMSE	$\sqrt{\frac{1}{N}\sum_{i=1}^{N}{\left({E}_{i}-{P}_{i}\right)}^{2}}$	
4	MAE	$\frac{1}{N}\sum_{i=1}^{N}\left|\frac{{E}_{i}-{P}_{i}}{{E}_{i}}\right|\times 100$	
5	NSEI	$1- \frac{{\sum }_{i=1}^{N}{({E}_{i}-{P}_{i})}^{2}}{{\sum }_{i=1}^{N}{({E}_{i}-\overline{P })}^{2}}$	
6	a20-index	$\frac{m20}{N}$	
7	RRMSE	$RRMSE= \frac{1}{\left|\overline{r }\right|}\sqrt{\frac{{\sum }_{i=1}^{N}{\left({E}_{i}- {P}_{i}\right)}^{2}}{N}}$	
8	*P*_*i*_	$\frac{RRMSE}{1+R}$	
9	OFA	$\left(\frac{{N}_{Training}- {N}_{Testing}}{N}\right){P}_{i,Training}+2\left(\frac{{N}_{Testing}}{N}\right){P}_{i,Testing}$	

### Artificial neural network

**Figure 4:**
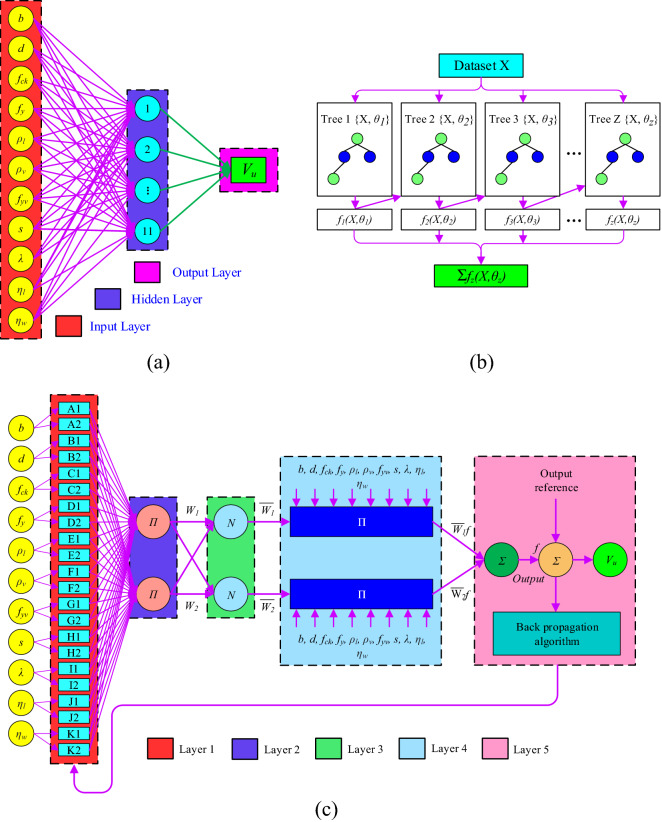
Architecture of ML models (**a**) ANN, (**b**) XGBoost and (**c**) ANFIS Model.

The study of biological neural links served as inspiration for ANN research. ANN algorithm is a "black box" that houses a massively parallel system with numerous processing components that are very good at information mining. The procedure inside the box enables a careful selection of the variables and a detailed investigation of their relationships^51,52^. In different ANN algorithms, a back-propagation network (BPN) is frequently used to solve engineering problems with the gradient descent technique to reduce errors. A typical BPN contains three layers: (a) input layer (IL), (b) hidden layer (HL), and (c) output layer (OL) as presented in Fig. 4a. The HL neurons are connected to each input neuron, which represents an individual input parameter. Depending on the kind of operation (linear or non-linear), these neurons sum the weighted values or apply the activation function after receiving information from the appropriate IL to produce the desired output. The extra node known as bias is present in both the HL and OLs.

Three layers of neurons are linked together by connections known as weights. To estimate the output of an ANN algorithm for a specific pattern, the biases and weights must be adequate. Each neuron that receives a numerical input from the preceding layer has its relevance determined by weighting variables. The shear strength of the CRCBs can be evaluated using Eq. (25):

25 $${V}_{u}= {f}_{\left(H-O\right)}\left(\sum_{i=1}^{N}{W}_{i\left(H-O\right)}{N}_{i}+{B}_{\left(H-O\right)}\right)$$

where, ${f}_{\left(H-O\right)}$ is the OL activation function as expressed in Eq. (26), ${W}_{i\left(H-O\right)}$ are the OL weights, ${N}_{i}$ are the input variables and can be obtained from Eq. (27), and ${B}_{\left(H-O\right)}$ is the output bias.

26 $${f}_{\left(H-O\right)}=purelin=f\left(x\right)=x$$

27 $${N}_{i}={f}_{\left(I-H\right)}\left(\sum_{i=1}^{N}{W}_{i\left(I-H\right)}{X}_{i}+{B}_{\left(I-H\right)}\right)$$

where, ${f}_{\left(I-H\right)}$ is activation function in the HL as expressed in Eq. (28), ${W}_{i\left(I-H\right)}$ is the HL weights, ${X}_{i}$ is the normalized input values, and ${B}_{\left(I-H\right)}$ is the HL biases.

28 $${f}_{\left(I-H\right)}= TanSig= \frac{2}{1-{e}^{-2z}}-1$$

**Figure 5:**
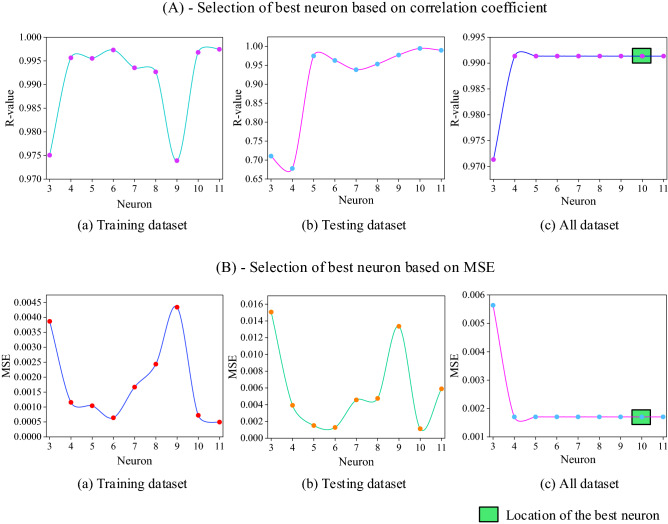
Selection of optimum neuron.

ANN has been trained from three neurons to eleven neurons. A trial and error process has been adopted to select the optimum neuron. On the basis of the R-value and MSE value the best-selected neuron is selected as shown in Fig. 5. The best neuron is chosen based on performance indicators. In the range of three to eleven neurons, neuron ten has the highest R-value and the lowest MSE value. The overall evaluation of neuron ten is acceptable. The correlation coefficient and MSE values of the training phase, testing phase, and whole phase is shown in Fig. 5.

The parrot-colored rectangular box depicts the location of the best neuron. The formulation to predict the shear strength of CRCBs is expressed in Eqs. (29) and (30).

29 $$\left[\begin{array}{c}{d}_{1}\\ {d}_{2}\\ {d}_{3}\\ {d}_{4}\\ {d}_{5}\\ {d}_{6}\\ {d}_{7}\\ {d}_{8}\\ {d}_{9}\\ {d}_{10}\end{array}\right]=tansig\left[\left(\begin{array}{ccccccccccc}-0.8071& 0.0620& -0.9229& 0.2073& 0.5096& -1.3520& 1.9673& 0.0969& -0.0353& 1.2739& 0.4922\\ 0.4208& -0.1356& -0.3379& -0.1556& 0.2532& -0.4656& 0.8207& -0.7836& 2.0054& -0.1553& -0.2239\\ -1.9789& -0.8337& 0.5156& -0.3473& 0.4251& -0.5391& -0.6441& 0.7573& -1.2279& 0.4585& 0.5222\\ 0.0980& -0.0709& 0.2388& 0.7224& -0.7378& -0.3546& -0.3905& 1.6784& -2.9510& 1.0597& -1.4663\\ -2.2629& -0.3706& 0.9284& 0.5114& 0.7825& -0.1382& -1.4820& 1.7856& -0.1469& -0.6652& 0.5620\\ 1.7002& -0.0002& -0.2672& 1.5503& 1.0243& 0.1634& 0.3901& 0.3129& 1.9112& -0.4058& 0.8284\\ 1.5318& -0.9390& 0.0097& -0.3094& -0.9207& 0.9510& -0.7813& 0.2750& -1.0490& -0.9694& 2.0092\\ 0.3404& -0.2200& 0.7990& -1.7213& 0.6330& 0.8612& -0.2832& -0.8524& -2.2531& -1.7455& 1.1284\\ 0.2358& 0.8619& 0.2756& 0.4233& 1.1082& -1.8456& 0.5682& -0.0741& 2.7171& 1.8584& -0.6170\\ -0.9259& -0.4344& -0.9707& 0.4044& 0.0502& -0.2438& 0.6536& 0.0834& -0.9066& -1.6718& -0.1058\end{array}\right)\times \left(\begin{array}{c}b\\ d\\ {f}_{ck}\\ {f}_{y}\\ {\rho }_{l}\\ {\rho }_{v}\\ {f}_{yv}\\ s\\ \lambda \\ {\eta }_{l}\\ {\eta }_{w}\end{array}\right)\right]+\left[\begin{array}{c}-1.9573\\ -1.2184\\ 0.9513\\ -1.8569\\ 1.7187\\ 0.4107\\ -1.7598\\ -1.3854\\ 1.0573\\ -1.9978\end{array}\right]$$

30 $$-0.1885{d}_{1}-0.4275{d}_{2}+0.4295{d}_{3}+0.5789{d}_{4}-0.7536{d}_{5}+0.9335{d}_{6}-0.4282{d}_{7}+0.5503{d}_{8}+0.4771{d}_{9}+0.4468{d}_{10}-0.3566$$

The values of ${d}_{1}$ to ${d}_{10}$ can be calculated using Eq. (29).

### Adaptive neuro-fuzzy inference system

ANFIS is the name of the hybrid neuro-fuzzy network that simulates complex systems. A fuzzy inference system (FIS), which is employed with an ANN, and Takagi–Sugeno rule type make up the majority of the ANFIS model. An adaptive and feed-forward network derives fuzzy rules from inputs using an ANFIS technique. A hybrid learning approach employs the fuzzy membership function (MF) parameters and looks for connections between the inputs and outputs based on the knowledge of expert systems. The basic architecture of the ANFIS model is presented in Fig. 4c. The ANFIS structure consists of five layers, namely, the “fuzzy layer”, “product layer”, “normalized layer”, “de-fuzzy layer”, and “total OL” (Fig. 4c). The formulation of each layer and complete description is available in the literature^53–55^.

**Layer 1:** All nodes in this layer are adaptive nodes. MFs like the Gaussian MF and generalized bell MF are employed as node functions.

**Layer 2:** Each node output in this layer displays the firing rate of a rule.

**Layer 3:** The normalized firing strength of each rule is represented by each node.

**Layer 4:** Each node in this layer is adaptive and has a node function that describes how the rules contributed to the final output.

**Layer 5:** The sum of all the rules outputs is computed by a single node.

The subtractive clustering approach and the grid partitioning method are used to choose the initial fuzzy model based on the fuzzy rules specified. The subtractive clustering approach is adopted in the development of the ANFIS model. Locating the cluster centres of the input–output data pairs is made easier by the cluster estimation approach. This in turn aids in the identification of the rules that are dispersed across the input–output space, as each cluster centre denotes the existence of a rule. Additionally, it aids in figuring out what the underlying premise parameters should be set to. This is crucial because, during the neural network training session, a starting value that is very near to the ultimate value will eventually force the model to quickly converge to that value^56^. The potentials of all the input and output data points are determined using the Euclidian distances between them and the other data points in this clustering approach.

**Figure 6:**
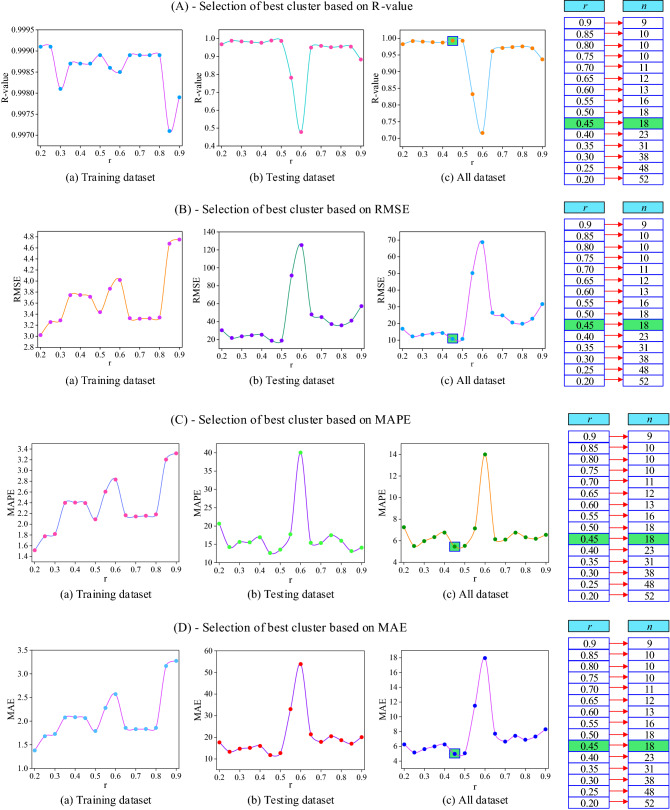
Selection of optimum cluster center (*r*) based on R and RMSE values. Selection of optimum cluster center (*r*) based on MAPE and MAE values.

**Figure 7:**
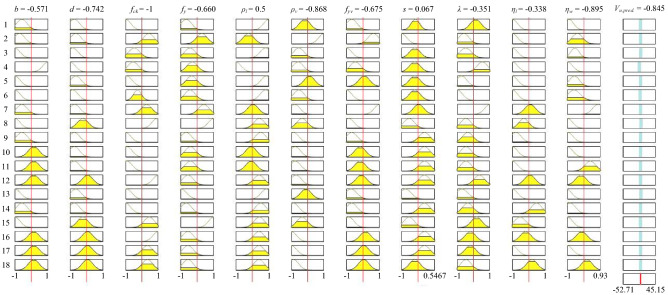
Rules of the established ANFIS model.

**Figure 8:**
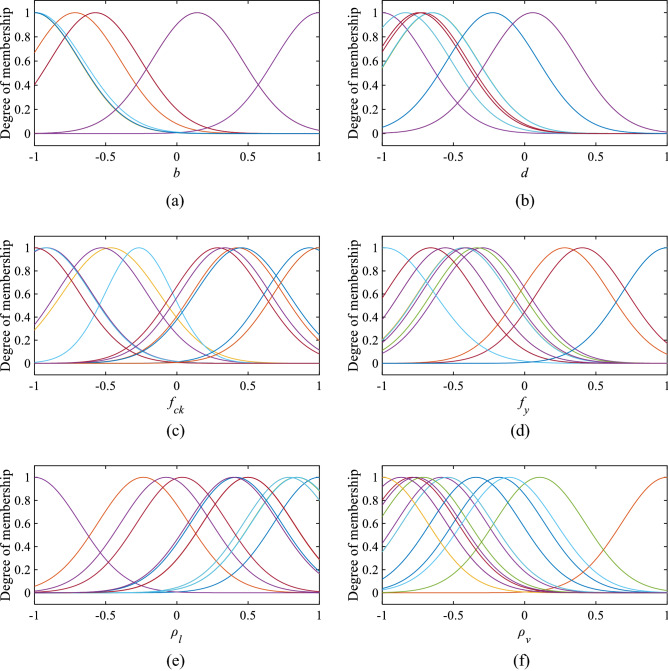

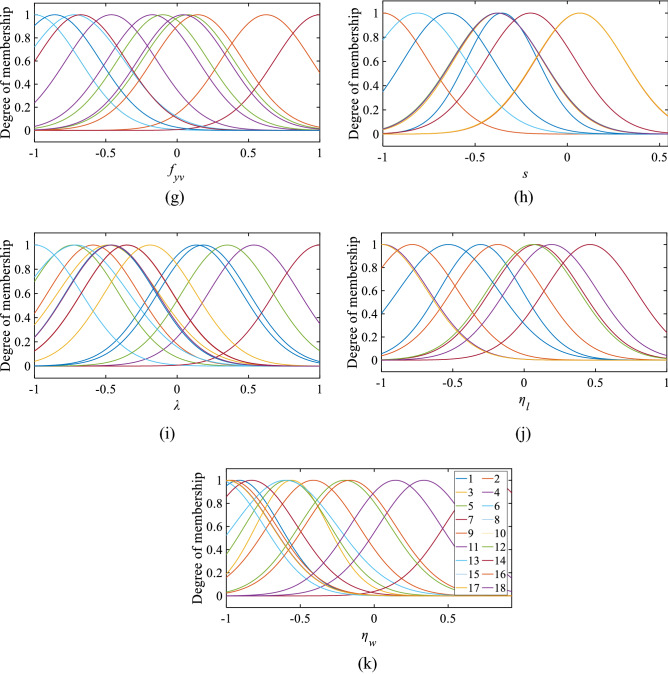
MF of the selected ANFIS model (**a**) *b*, (**b**) *d*, (**c**) *f*_*ck*_, (**d**) *f*_*y*_, (**e**) ${\rho }_{l}$, and (**f**) ${\rho }_{v}$, (g) *f*_*yv*_, (**h**) *s*, (**i**) $\lambda$, (**j**) ${\eta }_{l}$ and (**k**) ${\eta }_{w}$.

In the subtractive clustering approach, the squash factor, reject ratio, and accept ratio are taken as constant with values of 1.25, 0.5, and 0.15, sequentially. The cluster centre (r) is changed from 0.9 to 0.2 value. The best cluster centre is chosen based on performance indicators. In the range of 0.9–0.2 cluster centre, cluster centre 0.45 has the highest R-value and the lowest RMSE, MAPE, and MSE values. The overall evaluation of cluster centre 0.45 is acceptable. In Fig. 6, the performance of all the cluster centre is shown with a number of rules (*n*). The number of rules and MFs are in Figs. 7 and 8, respectively.

The shear predictions using the ANFIS model is expressed in Eq. (31).

31 $${V}_{u,pred.}= \frac{\sum_{i=1}^{n}{W}_{i}{Y}_{i}}{{\sum }_{i=1}^{n}{W}_{i}}$$

The values ${W}_{i}$ and ${Y}_{i}$ are expressed in Eqs. (32) and (33), respectively.

32 $${W}_{i}= \left[exp\left(-\frac{1}{2}{\left(\frac{b- {c}_{1}}{{\sigma }_{1}}\right)}^{2}\right)\right] \times \left[exp\left(-\frac{1}{2}{\left(\frac{d- {c}_{2}}{{\sigma }_{2}}\right)}^{2}\right)\right] \times \left[exp\left(-\frac{1}{2}{\left(\frac{{f}_{ck}- {c}_{3}}{{\sigma }_{3}}\right)}^{2}\right)\right] \times \left[exp\left(-\frac{1}{2}{\left(\frac{{f}_{y}- {c}_{4}}{{\sigma }_{4}}\right)}^{2}\right)\right]\times \left[exp\left(-\frac{1}{2}{\left(\frac{{\rho }_{l}- {c}_{5}}{{\sigma }_{5}}\right)}^{2}\right)\right] \times \left[exp\left(-\frac{1}{2}{\left(\frac{{\rho }_{v}- {c}_{6}}{{\sigma }_{6}}\right)}^{2}\right)\right] \times \left[exp\left(-\frac{1}{2}{\left(\frac{{f}_{yv}- {c}_{7}}{{\sigma }_{7}}\right)}^{2}\right)\right] \times \left[exp\left(-\frac{1}{2}{\left(\frac{s- {c}_{8}}{{\sigma }_{8}}\right)}^{2}\right)\right]\times \left[exp\left(-\frac{1}{2}{\left(\frac{\lambda - {c}_{9}}{{\sigma }_{9}}\right)}^{2}\right)\right]\times \left[exp\left(-\frac{1}{2}{\left(\frac{{\eta }_{l}- {c}_{10}}{{\sigma }_{10}}\right)}^{2}\right)\right]\times \left[exp\left(-\frac{1}{2}{\left(\frac{{\eta }_{w}- {c}_{11}}{{\sigma }_{11}}\right)}^{2}\right)\right]$$

where, $b$, $d$, ${f}_{ck}$, ${f}_{y}$, ${\rho }_{l}$, ${\rho }_{v}$, ${f}_{yv}$, $s$, $\lambda$, ${\eta }_{l}$, and ${\eta }_{w}$ are the input variables (normalized values), and $\sigma$ and *c* are the Gaussian MF parameters.

33 $${Y}_{i}=\boldsymbol{ }{k}_{1} b+ {k}_{2} d+ {k}_{3} {f}_{ck}+{k}_{4} {f}_{y}+ {k}_{5} {\rho }_{l}+ {k}_{6} {\rho }_{v}+ {k}_{7} {f}_{yv}+{k}_{8} s+ {k}_{9} \lambda + {k}_{10} {\eta }_{l}+ {k}_{11} {\eta }_{w}+{k}_{12}$$

The values of *k*_*1*_, to *k*_*12*_ are given in Table 3, and c and $\sigma$ values of input parameters are in Table 4.

**Table 3:** Coefficients of membership cluster plot (normalized values).

Rule no	${k}_{1}$	${k}_{2}$	${k}_{3}$	${k}_{4}$	${k}_{5}$	${k}_{6}$	${k}_{7}$	${k}_{8}$	${k}_{9}$	${k}_{10}$	${k}_{11}$	${k}_{12}$
1	0.5952	0.1915	− 0.8659	− 0.2932	0.3666	− 0.0850	0.7986	1.6790	0.7859	0.0832	0.6507	0.0010
2	0.1039	0.0932	− 0.0594	− 0.0392	0.0258	− 0.1442	− 0.0871	0.1397	0.0793	0.1397	− 0.1333	− 0.1397
3	− 3.0570	− 0.5361	1.2350	3.3750	− 1.2930	2.1300	− 2.0500	− 1.7170	5.3620	− 0.0709	0.1482	0.1249
4	− 4.1170	4.1170	− 0.0549	1.2150	4.1170	2.4120	1.8980	1.5550	− 2.2170	− 0.3409	− 19.7700	− 4.1170
5	0.0422	0.0269	0.0336	1.3010	− 0.0322	− 0.0205	− 0.0766	− 0.0441	0.0263	0.0402	− 0.0037	− 0.0399
6	1.1230	0.8158	− 3.4990	0.9940	− 0.9201	0.3232	0.9489	0.7169	− 5.6660	1.1250	− 0.7337	− 1.1240
7	0.1213	0.1525	0.0149	− 0.0858	− 0.0081	0.1676	− 0.2123	0.0425	− 0.2123	− 0.1178	0.0137	− 0.2123
8	− 0.9276	− 0.2090	− 4.7220	0.9276	0.3852	− 0.3173	− 0.7954	− 0.5978	2.6620	0.3315	− 0.9276	0.9276
9	0.3918	− 0.0266	− 0.7952	− 0.3876	− 0.1016	− 0.5806	0.2635	0.4002	0.1474	− 0.3483	2.5090	0.1854
10	0.3034	0.0745	0.1419	0.1586	− 0.2488	0.1242	0.2442	0.0143	0.0033	0.4008	− 0.1295	− 0.1128
11	− 0.0269	0.1290	0.1726	0.0773	0.0142	0.1332	0.0328	0.0139	0.0868	0.1890	0.0543	− 0.1890
12	− 0.0516	− 0.0204	0.1005	0.2010	− 0.1431	0.258	− 0.0219	− 0.0040	− 0.1024	0.0119	0.0787	− 0.3611
13	0.6952	− 0.8565	− 2.0150	0.0886	1.6950	0.4855	5.0140	0.9109	1.8460	− 1.1180	0.1374	1.1200
14	0.0915	0.1188	0.1601	0.1057	− 0.0802	0.1390	0.1081	− 0.0106	0.0562	− 0.0354	0.0683	− 0.1601
15	0.2795	0.0630	− 2.3860	− 0.2795	− 0.1161	0.0956	0.2397	0.1802	2.0360	− 2.3620	0.2795	− 0.2795
16	− 0.0359	− 0.0154	− 0.3115	0.1339	− 0.0952	− 0.1161	− 0.1673	− 0.2732	0.0083	− 0.3444	− 0.0251	− 0.2414
17	0.4053	0.5089	− 0.2638	0.1986	− 0.1725	0.6489	− 0.0627	− 0.0690	− 0.1327	− 0.1244	− 0.1952	− 0.1104
18	− 0.0230	− 0.0091	− 0.2675	0.0896	− 0.0638	0.1413	0.0389	0.0937	0.0811	− 0.1120	− 0.1021	− 0.1610

**Table 4:** Parameters of Gaussian MF (normalized values).

MF	*b*		*d*		*f*_*ck*_		*f*_*y*_		*ρ*_*l*_		*ρ*_*v*_		*f*_*yv*_		*s*		*λ*		*ƞ*_*l*_		*ƞ*_*w*_	
	${c}_{1}$	${\sigma }_{1}$	${c}_{2}$	${\sigma }_{2}$	${c}_{3}$	${\sigma }_{3}$	${c}_{4}$	${\sigma }_{4}$	${c}_{5}$	${\sigma }_{5}$	${c}_{6}$	${\sigma }_{6}$	${c}_{7}$	${\sigma }_{7}$	${c}_{8}$	${\sigma }_{8}$	${c}_{9}$	${\sigma }_{9}$	${c}_{10}$	${\sigma }_{10}$	${c}_{11}$	${\sigma }_{11}$
1	− 0.5717	0.3185	− 0.7418	0.3182	− 0.9998	0.3186	− 0.6600	0.3183	0.9999	0.3182	− 0.1813	0.3187	− 0.6753	0.3186	− 0.3532	0.1950	0.1348	0.3189	− 1.0000	0.3182	− 0.9123	0.2763
2	− 0.7143	0.3182	− 0.6479	0.3182	0.4255	0.3182	0.2808	0.3182	− 0.2354	0.3182	1.0000	0.3182	0.6239	0.3182	− 1.0000	0.2461	− 0.5892	0.3182	− 1.0000	0.3182	− 0.4126	0.3071
3	− 1.0013	0.3165	− 0.8361	0.3181	− 0.4639	0.3392	− 0.4317	0.3204	0.7878	0.3178	− 0.5412	0.3183	− 1.0010	0.3174	− 0.3755	0.2416	− 0.4842	0.3514	− 1.0001	0.3184	− 0.5561	0.2433
4	1.0000	0.3182	− 1.0000	0.3182	− 0.5282	0.3182	− 0.2951	0.3182	− 1.0000	0.3182	− 0.5857	0.3182	− 0.4610	0.3182	− 0.3778	0.2461	0.5385	0.3182	− 0.9885	0.3182	− 0.9969	0.3071
5	− 1.0000	0.3182	− 0.6479	0.3182	− 0.9198	0.3182	− 0.3349	0.3181	0.8397	0.3182	0.1049	0.3184	0.0755	0.3218	− 0.3791	0.2484	− 0.7297	0.3182	− 1.0000	0.3182	− 0.5862	0.2890
6	− 1.0000	0.3182	− 0.8356	0.3183	− 0.2665	0.2370	− 0.4302	0.3178	0.7884	0.3182	− 0.5415	0.3185	− 0.9998	0.3184	− 0.3782	0.2467	− 0.9963	0.3242	− 1.0000	0.3182	− 0.6014	0.3506
7	− 0.5714	0.3182	− 0.7183	0.3182	0.2866	0.3182	0.4039	0.3182	0.0381	0.3182	− 0.7895	0.3182	1.0000	0.3182	− 0.2000	0.2461	1.0000	0.3182	0.0846	0.3182	0.7819	0.3071
8	− 1.0000	0.3182	− 0.2254	0.3182	0.9312	0.3156	1.0000	0.3182	0.4153	0.3182	− 0.3421	0.3182	− 0.8576	0.3182	− 0.6444	0.2461	− 0.4663	0.3153	− 0.3047	0.2893	− 1.0000	0.3071
9	− 0.5716	0.3184	− 0.7418	0.3182	− 0.9998	0.3185	− 0.6600	0.3183	0.4995	0.3170	− 0.8684	0.3185	− 0.6753	0.3183	0.0677	0.2441	− 0.3522	0.3170	− 0.7842	0.3177	− 0.9672	0.3056
10	0.1428	0.3184	− 0.7418	0.3182	− 0.9134	0.3182	− 0.4089	0.3182	− 0.0750	0.3183	− 0.7632	0.3182	− 0.1738	0.3183	0.0667	0.2461	− 0.4595	0.3182	− 1.0001	0.3182	− 1.0000	0.3071
11	0.1429	0.3182	− 0.7418	0.3182	− 0.9134	0.3182	− 0.4089	0.3182	− 0.0751	0.3182	− 0.7632	0.3182	− 0.1738	0.3182	0.0667	0.2461	− 0.4595	0.3182	− 1.0000	0.3182	0.3362	0.3071
12	0.1429	0.3182	0.0563	0.3182	1.0000	0.3182	− 0.5567	0.3182	0.3965	0.3182	− 0.7105	0.3182	− 0.0997	0.3182	0.0667	0.2461	0.3514	0.3182	0.0615	0.3182	− 0.1996	0.3071
13	− 0.9889	0.3329	− 0.6506	0.3190	− 0.9217	0.3189	− 0.9809	0.3470	0.8440	0.3204	− 0.1100	0.3275	− 0.7191	0.3472	− 0.8120	0.2639	− 0.7095	0.3695	− 1.0000	0.3182	− 1.0460	0.2998
14	− 0.5714	0.3182	− 0.7418	0.3182	− 1.0000	0.3182	− 0.6601	0.3182	0.5002	0.3182	− 0.8684	0.3182	− 0.6752	0.3182	0.0667	0.2461	− 0.3514	0.3182	0.4616	0.3180	− 0.8292	0.3071
15	− 1.0000	0.3182	− 0.2254	0.3182	0.4545	0.3261	1.0000	0.3182	0.4153	0.3182	− 0.3421	0.3182	− 0.8576	0.3182	− 0.6444	0.2461	0.1841	0.3177	− 0.5307	0.3486	− 1.0000	0.3071
16	0.1429	0.3182	0.0563	0.3182	1.0000	0.3182	− 0.5567	0.3182	0.3965	0.3182	− 1.0000	0.3182	0.1453	0.3182	0.0667	0.2461	− 0.1892	0.3182	− 0.1846	0.3182	− 0.1626	0.3071
17	0.1429	0.3182	0.0563	0.3182	0.3376	0.3182	− 0.5567	0.3182	0.3965	0.3182	− 1.0000	0.3182	0.0427	0.3182	0.0667	0.2461	− 0.1892	0.3182	− 1.0000	0.3182	− 1.0000	0.3071
18	0.1429	0.3182	0.0563	0.3182	0.3376	0.3182	− 0.5567	0.3182	0.3965	0.3182	− 0.8684	0.3182	0.0427	0.3182	− 0.3778	0.2461	− 0.4595	0.3182	0.1923	0.3182	0.1440	0.3071

### Decision tree (DT)

A decision tree is a structure that resembles a flowchart and is used to illustrate a decision-making process. It is a kind of technique for supervised learning that may be applied to both classification and regression applications. In order to create subsets (or "leaves") that are as homogeneous as feasible with regard to the target variable, the dataset is recursively split into subsets based on the values of the input features.

The "root" node of the tree, which represents the complete dataset, is the first node in the tree. Then, based on a selected feature and a threshold value, the root node is divided into two or more child nodes. Recursive splitting occurs on each child node until a halting requirement is satisfied. For instance, the tree can be terminated when a node reaches a predetermined threshold for data points or when all of the data points in a node belong to the same class.

Each leaf node of the tree represents a class label (in the case of a classification problem) or a predicted value, and each internal node of the tree represents a test on an input characteristic (in the case of a regression problem)^57^. A set of choices that result in a particular conclusion are represented by the route from the root to a leaf node. A decision tree can be used for prediction by going from the root to a leaf node and selecting the class label or predicted value linked to that leaf node. Decision trees have a number of benefits, one of which is their readability and comprehension due to the clear and logical representation of the decision-making process. They may, however, be prone to overfitting if they are not appropriately trimmed or regularized.

The simplicity of understanding and visualization, ease of data pre-processing, and insensitivity to outliers are all advantages of DT over other ML models^58^. In this study, both tenfold cross-validations along with grid search are used to optimize the decision tree. The values of tuning hyper-parameters are auto, 10, 2, 5, and 100 for parameters of max features, max depth, min samples leaf, min samples split, and max-leaf nodes, respectively.

### eXtreme gradient boosting (XGBoost)

XGBoost is a highly effective and scalable ML algorithm for tree boosting and has been widely employed in various domains to produce cutting-edge outcomes on specific data difficulties. The gradient boosting framework is optimized in XGBoost, which is created to be extremely effective, adaptable, and portable^59^. The basic task of the XGBoost method is to optimize the value of the objective function, which consists of the regularisation term and the loss function. Although the regularisation term serves to smooth the final learned weights to limit overfitting, the loss function, which calculates the difference between the estimated and actual label for a given training sample, minimizes the error of the entire model. A few XGBoost tuning settings have a significant impact on the model's performance and training efficiency. The learning rate, maximum depth of a tree, minimal sum of instance weight, subsample, and the number of boosting iterations are the some hyper tuning parameters. The basic architecture of the XGBoost model is depicted in Fig. 4b. The pseudocode of the XGBoost algorithm is mentioned below:
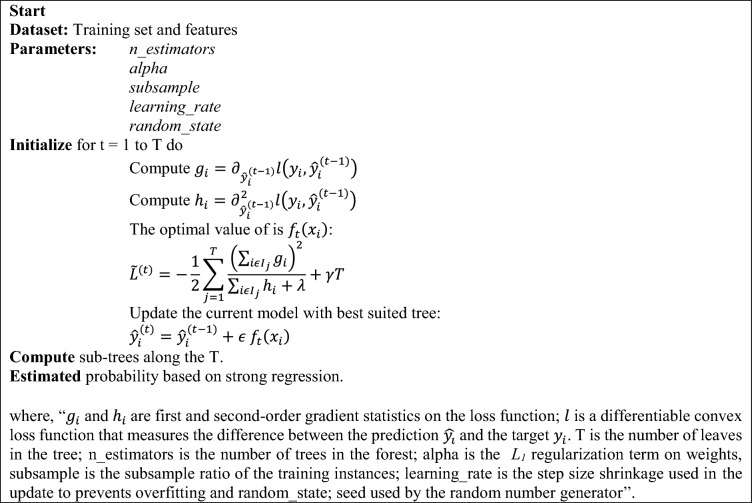


Compute the gradient ${g}_{i}$ and hessian ${h}_{i}$ of the loss function $l\left({y}_{i},\right){\widehat{{y}_{i}}}^{\left(t-1\right)}$ with respect to the current model's predictions ${\widehat{y}}_{i}^{\left(t-1\right)}$. Solve for the optimal value of the new tree ${f}_{t}\left({x}_{i}\right)$ by minimizing an approximation of the negative gradient, this step is known as the line search. This is done by computing the ${\widetilde{L}}^{(t)}$ function which is a combination of the gradient and hessian of the loss function and *T*. Update the current model by adding the new tree ${f}_{t}\left({x}_{i}\right)$ to the ensemble, with a step size of *ϵ*, ${\widehat{{y}_{i}}}^{\left(t\right)}$= ${\widehat{y}}_{i}^{\left(t-1\right)}+\upepsilon {f}_{t}\left({x}_{i}\right)$. The iteration process starts and stops when the required criteria are met, such as a maximum number of trees or a minimum improvement in the error. In the end, a final ensemble of decision trees is used to make predictions on new data.

Grid search, randomized search, and tenfold cross-validation are used to optimize the XGBoost hyperparameters. Through a random search, the initial hyperparameters are obtained. Then, using a grid-search approach, the resulting hyperparameters are optimized. Ten folds are randomly selected from the training dataset. Nine folds are employed in this technique for model training, and one fold is used for performance evaluation. The cross-validation procedure was then carried out ten times, using the validation data from each of the ten subsamples exactly once each time^60^. The values of the grid and random search hyperparameters used in the XGBoost model are 100, 10, 0.9, 0.4, and 123 for parameters of *n* estimators, alpha, subsample, learning rate, and random state, respectively.

## Results and discussion

The results and discussion section is categorized into four subsections. In the first subsection, the results of the analytical models are explained with scatter plots, 2D kernel plots, and absolute error plots. The findings of the ANN, ANFIS, DT, and XGBoost models are also discussed graphically in the second part. Additionally, the line plot of the experimental, predicted, and error data is also utilized to make the produced models more visible. The comparison between the analytical and ML-based models is explained in the discussion section with the violin and Taylor plot. The influence of single parameters on the shear strength of the CRCBs is explained in the last subsection.

### Outcomes of analytical models

Six analytical models and one existing ML (Gradient Boosted Regression Trees (GBRT)) model have been used to assess the performance of the ML models. When comparing analytical models, the correlation coefficient of Lu et al.'s model is the highest and the values of the models by Xu and Niu, Huo, Zhao and Jin, Yu, and Li et al. decreasing sequentially. However, Huo's model has the lowest MAPE value and this value is 14.61% lower than that of Lu et al.'s model. The other performance metrics of Lu et al.'s confirmed the accuracy of this model compared to other analytical models. Table 5 shows the values for each performance metric.

**Table 5:** Results of analytical models and existing ML model.

S. no	Model	R	MAPE (%)	MAE (kN)	RMSE (kN)	NS	a20-index
1	Xu and Niu	0.8365	32.9710	38.8748	51.3782	0.6781	0.2286
2	Yu	0.7577	37.0414	44.6898	63.6309	0.5062	0.3071
3	Huo	0.8255	30.2997	37.1567	52.9301	0.6583	0.2929
4	Zhao and Jin	0.8099	62.9772	67.8452	86.0807	0.0964	0.1286
5	Li et al	0.7585	41.6435	51.1521	72.8369	0.3530	0.1571
6	Liu et al	0.8689	35.4832	36.5377	48.4388	0.7139	0.3500
7	Fu and Feng (GBRT)^62^	0.9889	–	9.5800	14.0500	–	–

**Figure 9:**
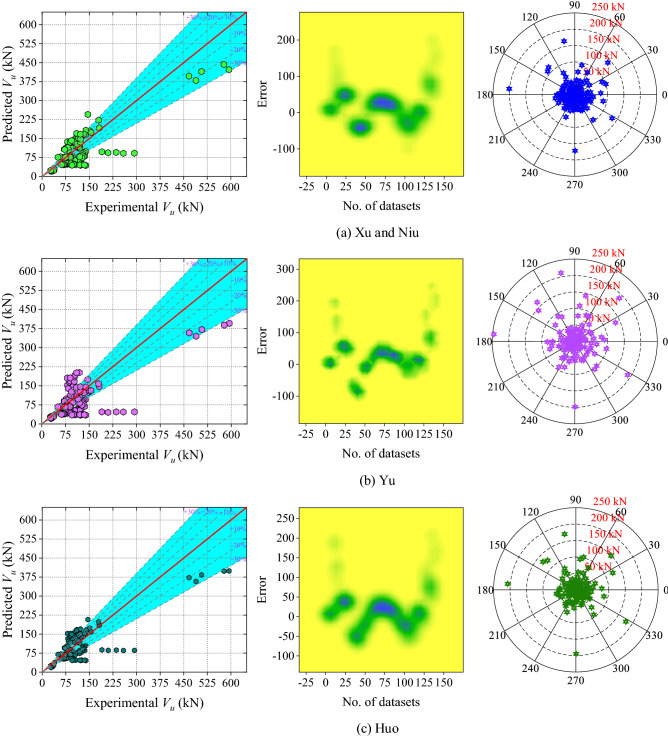

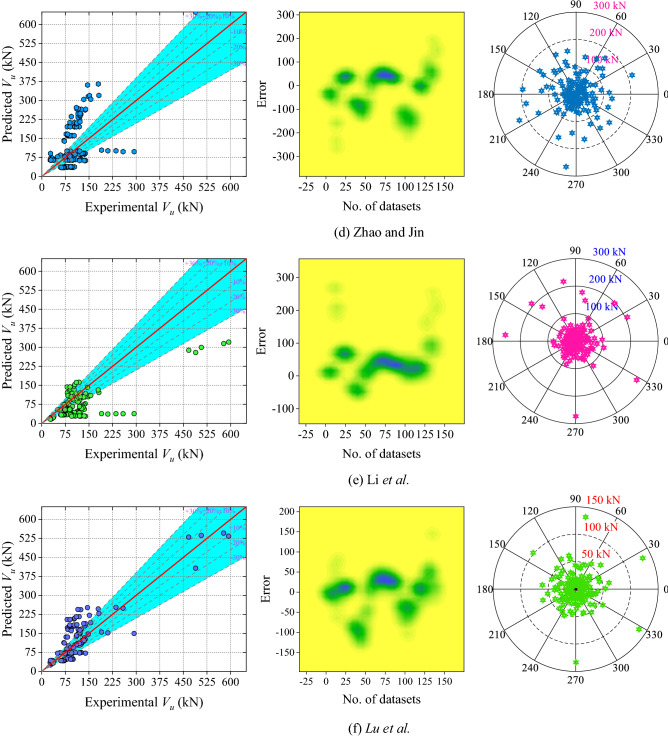
Results of the analytical models (**a**) Xu and Niu, (**b**) Yu, (c) Huo, (**d**) Zhao and Jin, (**e**) Li et al., and (**f**) Lu et al.

The scatter plot (Fig. 9 (left-side), 2D kernel density (Fig. 9 (middle-plot) and absolute error plot (Fig. 9 (right-side) of all the analytical models are shown in Fig. 9. In Huo, Liu et al., Yu, Xu and Niu, Li et al., and Zhao and Jin model’s 55%, 52.14%, 49.29%, 46.42%, 36.42%, and 22.89% data lie in the range of − 30 to + 30 kN, respectively. The range of the error of the above mentioned analytical model is − 100 to 201.28 kN, − 99.50 to 246.13 kN, − 71.41 to 207.89 kN, − 268.43 to 195.69 kN, − 62.42 to 273.36 kN and − 129.27 to 144.10 kN. According to the absolute error plot (Fig. 9 (right-side)), in the model of Liu et al., approximately 80% of the dataset is inside the 50 kN error limit. Therefore, it can be inferred that the Liu et al. model performs well in comparison to other analytical models.

### Outcomes of XGBoost, DT, ANFIS, and ANN models

In all the models, the dataset is divided into two categories: (i) Training (70%), and (ii) Testing (30%) dataset. The R-values of the ANN training and testing dataset are 0.9908, and 0.9962, sequentially. The MAPE, RMSE, MAE, NSEI, and a20-index of the whole dataset (ANN) are 7.4703%, 12.2962 kN, 7.0135 kN, 0.9809, and 0.9357, respectively. The overfitting value of the ANN model is 0.0567 as shown in Table 6. In the ANFIS model, the R-value of the training and testing dataset is 0.9987 and 0.9894, respectively. The MPAE value of the ANFIS model is lower than the ANN model which is 5.4623%. Similarly, the MAPE values of the ANFIS model is 14.58% less than the DT model. The overall MAE and RMSE values of the ANFIS models is also less than the ANN and DT models. The ANFIS model has a higher NSEI and an a20-index than the ANN and DT models. The overfitting values of the ANFIS and ANN models are very close to each other. The MAPE value of the XGBoost model for the whole dataset is minimal which is 0.0459% and R-value is approximately equal to one. The a20-index and NS index also approaches to the value of one. The overfitting value of the XGBoost model is 0.0021, as shown in Table 6.

**Table 6:** Results of ANN, ANFIS, DT, and XGBoost model.

S. no	Model		R	MAPE (%)	MAE (kN)	RMSE (kN)	NS	a20-index	*P*_*i*_	OFA
1	ANN	Training	0.9908	5.2482	5.7648	10.1523	0.9812	0.9489	0.0437	0.0567
		Testing	0.9962	11.1211	9.5234	14.5629	0.9890	0.9524	0.0580	
		All	0.9905	7.4703	7.0135	12.2962	0.9809	0.9357	0.0530	
2	ANFIS	Training	0.9987	2.3935	2.0639	3.7146	0.9975	1	0.0159	0.0551
		Testing	0.9894	12.623	11.755	18.8090	0.9741	0.8571	0.0813	
		All	0.9933	5.4623	4.9713	10.7610	0.9854	0.9571	0.0463	
3	DT	Training	0.9905	4.8066	5.8488	12.3964	0.9720	0.9796	0.0534	0.0778
		Testing	0.9898	10.0982	11.2110	21.7765	0.9654	0.8810	0.0941	
		All	0.9899	6.3940	7.4575	15.8062	0.9685	0.9500	0.0681	
4	XGBoost	Training	0.9999	0.0542	0.1349	0.5890	0.9999	1	0.0025	0.0012
		Testing	0.9999	0.0267	0.0291	0.0603	1	1	0.0003	
		All	0.9999	0.0459	0.1031	0.4939	0.9999	1	0.0021	

**Figure 10:**
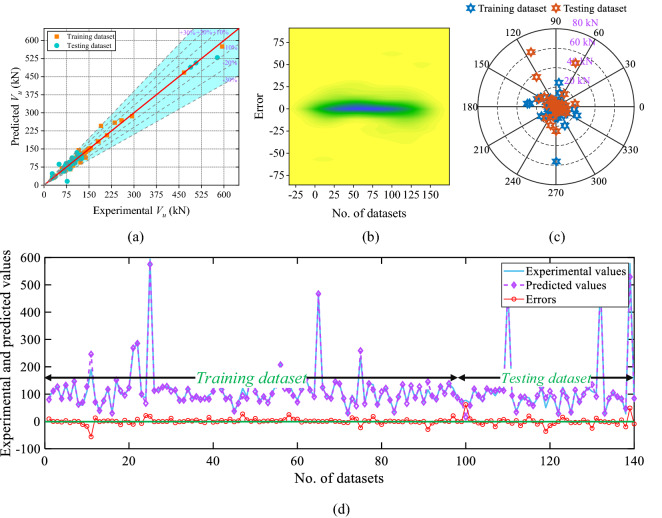
Results of the ANN model (**a**) scatter plot, (**b**) 2D Kernel density plot, (**c**) absolute error plot, and (**d**) line plot of the experimental and predicted values with errors.

The scatter plot, 2D kernel plot, absolute error plot and line plot of the ANN model is presented in Fig. 10a–d, sequentially. According to the scatter plot (Fig. 10a), only 25.72% of values directly lie over the fitting line whereas 80% of the values are inside the 10 kN absolute error limit. As per Fig. 10b,c, the error range is between − 36.39 and 61.60 kN. The line plot of the measured and predicted value with the distribution of the errors is presented in Fig. 10d.

**Figure 11:**
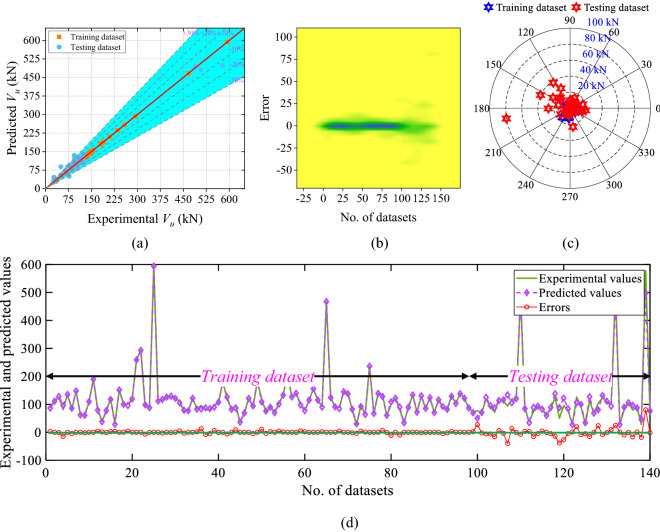
Results of the ANFIS model (**a**) scatter plot, (**b**) 2D Kernel density plot, (**c**) absolute error plot, and (**d**) line plot of the experimental and predicted values with errors.

Figure 11 shows the scatter plot, 2D kernel plot, absolute error plot, and line plot of the ANFIS model. According to the scatter plot (Fig. 11a), 45.72% dataset lie over the fitting line, and 87.14% of values are inside the 10 kN absolute error limit. The range of the error between − 40.24 and 80.19 kN is shown in Fig. 11b,c. The line plot of the phases of the train and test dataset of the predicted and experimental values is displayed in Fig. 11d.

**Figure 12:**
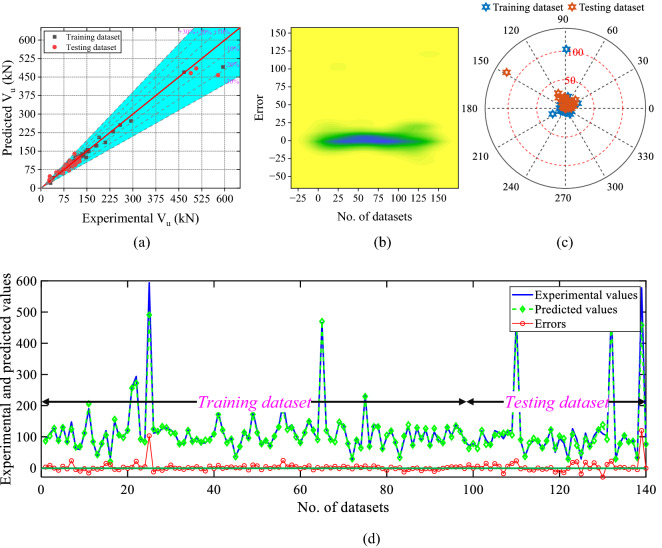
Results of the DT model (**a**) scatter plot, (**b**) 2D Kernel density plot, (**c**) absolute error plot, and (**d**) line plot of the predicted and experimental values with errors.

The scatter plot, 2D kernel plot, absolute error plot, and line plot of the XGBoost model is shown in Fig. 12. According to Fig. 12a, 13.57% of the dataset lie over the fitting line, and 82.14% of values inside the 10 kN absolute error limit. According to the 2D kernel plot (Fig. 12b) the range of the errors is between − 29.13 and 120.18 kN. Figure 12c,d show the absolute error and line plot of the developed DT model.

**Figure 13:**
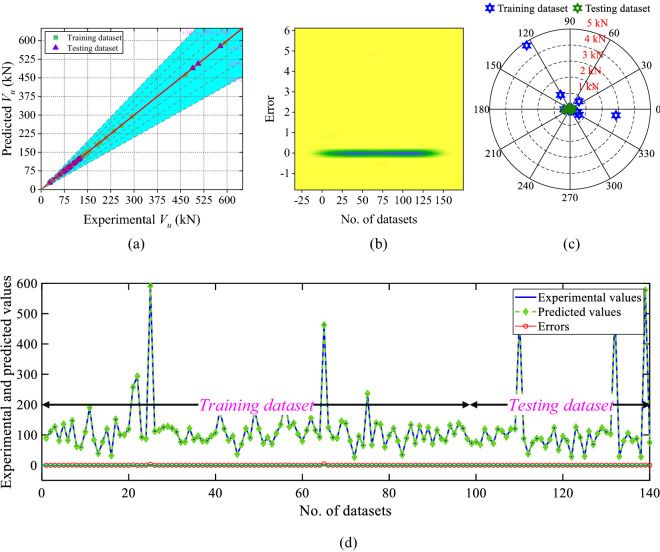
Results of the XGBoost model (**a**) scatter plot, (**b**) 2D Kernel density plot, (**c**) absolute error plot, and (**d**) line plot of the predicted and experimental values with errors.

Similarly, the scatter plot, 2D kernel plot, absolute error plot, and line plot of the XGBoost model is shown in Fig. 13. According to Fig. 13a, 97.86% dataset lie over the fitting line, and 99.29% of values inside the 2.88 kN absolute error limit. According to the 2D kernel plot (Fig. 13b) the range of the errors is between − 0.49 and 4.80 kN. Figure 13c,d show the absolute error and line plot of the developed XGBoost model.

The developed XGBoost model has greater performance and reliability when compared to ANN ANFIS, and DT models, according to performance metrics and graphical representations.

### Discussion

The results of the developed ML models have been compared with analytical models and existing ML-based models (Fu and Feng^61^). The R-value of the XGBoost model is 15.07%, 1.11%, 0.95%, 0.66%, and 1.01% higher than Lu et al*.*, Fu and Feng, ANN, ANFIS, and DT models, sequentially. Similarly, the NSEI and a20-index of the XGBoost model is 40.06%, 1.94%, 1.47%, and 3.24% and 185.71%, 6.87%, 4.48%, and 5.26% higher than Lu et al., ANN, ANFIS and DT models, respectively. On the other hand, the MAPE, RMSE, and MAE values of the XGBoost model is the lowest as shown in the last row of Table 7. The overfitting value of the XGBoost model is 97.88% 97.82%, and 98.46% lower than ANN, ANFIS, and DT models, respectively.

**Table 7:** Comparison of analytical and existing ML model with developed ML models.

S. no	Model	R	MAPE (%)	MAE (kN)	RMSE (kN)	NS	a20-index	*P*_*i*_	OFA
1	Liu et al	0.8689	35.4832	36.5377	48.4388	0.7139	0.3500	–	–
2	Fu and Feng (GBRT)^61^	0.9889	–	9.5800	14.0500	–	–	–	–
3	ANN	0.9905	7.4703	7.0135	12.2962	0.9809	0.9357	0.0530	0.0567
4	ANFIS	0.9933	5.4623	4.9713	10.7610	0.9854	0.9571	0.0463	0.0551
5	DT	0.9899	6.3940	7.4575	15.8062	0.9685	0.9500	0.0681	0.0778
6	XGBoost	0.9999	0.0459	0.1031	0.4939	0.9999	1	0.0021	0.0012

**Figure 14:**
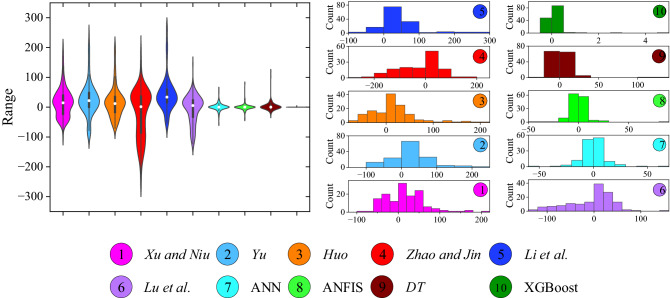
Performance comparison of analytical and ML-based models with violin and multi-histogram plot.

**Figure 15:**
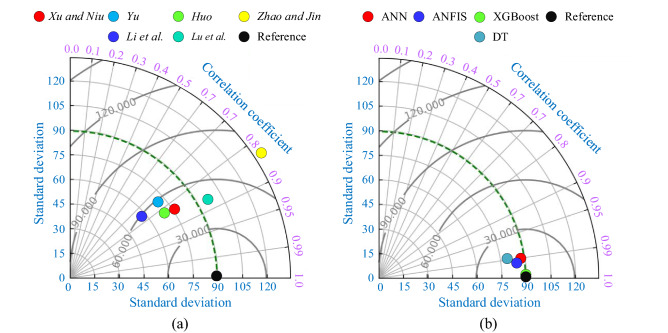
Taylor graph of (**a**) analytical models and (**b**) ANN, ANFIS, DT, and XGBoost models.

The violin plot and multi-histogram of all the analytical and developed ML models is shown in Fig. 14. From Fig. 14, it is clearly depicted that the accuracy of the XGBoost model is higher as compared to other models (analytical, ANN, ANFIS and DT). The Taylor diagrams of the analytical and ML models are shown in Fig. 15a,b, respectively. Taylor diagram is the graphical representation of the predicted values in relation to the original data. Taylor diagram plotted between the R, RMSE, and standard deviation. In Fig. 15a, two analytical models (Zhao and Lu et al.) crossed the reference line of the standard deviation, and Xu and Niu, Huo, and Liu et al. models lie below the 60 kN RMSE value. On the other hand, in Fig. 15b, the XGBoost model directly lies over the reference line of the original dataset. This ensures the reliability and precision of the XGBoost model among all the analytical and ML models.

### Feature importance

Lundberg and Lee developed a new technique to interpret black-box models called SHAP^62^. The SHAP is defined as “SHapley Additive exPlanations”. SHAP method uses game theory to characterize how well a machine-learning model performs.

**Figure 16:**
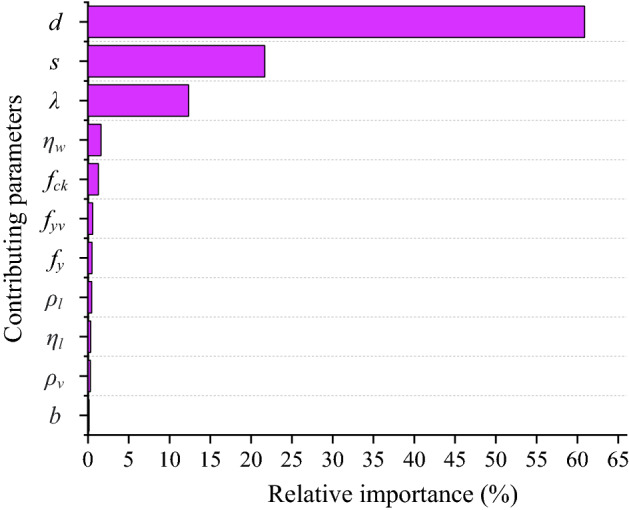
Feature importance.

It is crucial to carry out a variety of studies that are AI-based, adaptable, and capable of doing well on a variety of data. Through assessing its reliance on physical processes, sensitivity analysis (SA) and parametric studies help to confirm the robustness, effectiveness, and reliability of the generated MEP models. The influence of the individual parameter is shown in Fig. 16. According to the best-fitted model, the width of the beam, stirrups spacing, and the *a/d* is the most influencing factor with values of 60.86%, 21.67%, and 12.33% affect the shear capacity. The degree of corrosion of stirrups is only a 1.59% impact on the shear strength of the CRCBs.

## Conclusions

Estimating the shear capacity of the CRCBs is a very challenging issue in the civil engineering sector. To neutralize this issue, four ML-based algorithms (ANN, ANFIS, DT, and XGBoost) have been developed. The considered parameters that can influence the shear strength of the CRCBs are the width of the beam, the effective depth of the beam, CS of concrete, yield strength of steel, percentage of longitudinal steel, percentage of stirrups steel, yield strength of stirrups, stirrups spacing, *a/d*, corrosion degree of longitudinal steel, and corrosion degree of stirrups. Following is a summary of the conclusions drawn from the results of the analysis:

- Among analytical models, the prediction accuracy of Lu et al*.* is highest based on the performance metrics. The R-value and MAE of the Liu et al. model are 0.8689 and 36.54 kN, respectively.
- A single hidden layer with ten neurons has been used in the ANN and the model shows the good accuracy of the developed model. The R-value of the training, and testing, data is 0.9908, and 0.9962, sequentially. The MAPE value of the whole dataset is 7.47%.
- With a cluster radius 0.45 and eighteen rules, the performance of the ANFIS model is good. The R-value of the training and testing dataset is 0.9987 and 0.9894, respectively. The MAPE, MAE, and RMSE of the whole dataset are 5.46%, 4.97 kN, and 10.76 kN, respectively.
- The correlation coefficient of the DT model for the whole dataset is 0.9899. In addition, the error performance metrics of the DT model are higher than the ANFIS model.
- The correlation coefficient of the training and testing dataset of the XGBoost model is 0.9999 and 0.9999, respectively. The MAPE, MAE, and RMSE values of the whole dataset are 0.05%, 0.10 kN, and 0.49 kN, respectively.
- The excellent effectiveness of the XGBoost model in calculating the shear capacity of CRCBs was also shown, along with Taylor's graphical representation and violin plot.
- The developed model is very flexible and robust for engineers, requiring relatively few trial experiments. As a result, it saves more time and money throughout the CRCB strengthening process.

In addition to the experimental data gathered for this study, further research should utilize larger datasets. A GUI that enables interactive button-based task execution is also necessary to aid users in the practical and design interpretation of the shear capacity estimation^28^. Therefore, to develop multi-dimensional validation and improve the methodology employed in this work, the aforementioned elements should be taken into account and dealt with in later investigations.

## Supplementary Information

Supplementary Information.

## Data Availability

All data generated or analysed during this study are included in this published article (and its supplementary information file).

## References

[1] Fang C, Lundgren K, Plos M, Gylltoft K (2006). Bond behaviour of corroded reinforcing steel bars in concrete. Cem. Concr. Res..

[2] Xu T, Li J (2019). Experimental investigations of failure modes of reinforced concrete beams without web reinforcement. Eng. Struct..

[3] Xu S, Niu D (2004). The shear behavior of corroded simply supported reinforced concrete beam. J. Build. Struct.

[4] Yu, F. The test research and analysis on the shear strength of diagonal section in corroded reinforced concrete beam, 455 Master’s thesis. *Hohai University, China* **456** (2005).

[5] Huo Y (2007). Research on shear capacity of simply supported concrete beam with corroded reinforcement.

[6] Zhao Y-X, Jin W-L (2008). Analysis on shearing capacity of concrete beams with corroded stirrups. J. Zhejiang Univ. Eng. Sci..

[7] Shi-bin L, Xin Z (2011). Analysis for shear capacity of reinforced concrete beams with corrosion stirrups. J. Eng. Mech..

[8] Higgins, C. *et al.* Shear capacity assessment of corrosion-damaged reinforced concrete beams (Oregon. Dept. of Transportation. Research Unit, 2003).

[9] Webster, M. P. *The assessment of corrosion-damaged concrete structures*, University of Birmingham, (2000).

[10] Xue, X., Seki, H. & Chen, Z. in *Proceedings of the Thirteenth East Asia-Pacific Conference on Structural Engineering and Construction (EASEC-13).* C-6–2 (The Thirteenth East Asia-Pacific Conference on Structural Engineering).

[11] Khan I, François R, Castel A (2014). Experimental and analytical study of corroded shear-critical reinforced concrete beams. Mater. Struct..

[12] Khan NM (2022). Application of machine learning and multivariate statistics to predict uniaxial compressive strength and static Young’s modulus using physical properties under different thermal conditions. Sustainability.

[13] Nazar S (2022). Development of the new prediction models for the compressive strength of nanomodified concrete using novel machine learning techniques. Buildings.

[14] Kovačević M, Lozančić S, Nyarko EK, Hadzima-Nyarko M (2022). Application of artificial intelligence methods for predicting the compressive strength of self-compacting concrete with class F fly ash. Materials.

[15] Czarnecki S, Hadzima-Nyarko M, Chajec A, Sadowski Ł (2022). Design of a machine learning model for the precise manufacturing of green cementitious composites modified with waste granite powder. Sci. Rep..

[16] Asteris PG, Skentou AD, Bardhan A, Samui P, Pilakoutas K (2021). Predicting concrete compressive strength using hybrid ensembling of surrogate machine learning models. Cement Concr. Res..

[17] Rathakrishnan V, Beddu S, Ahmed AN (2022). Predicting compressive strength of high-performance concrete with high volume ground granulated blast-furnace slag replacement using boosting machine learning algorithms. Sci. Rep..

[18] Cai R (2020). Prediction of surface chloride concentration of marine concrete using ensemble machine learning. Cement Concr. Res..

[19] Taffese WZ, Espinosa-Leal L (2022). Prediction of chloride resistance level of concrete using machine learning for durability and service life assessment of building structures. J. Build. Eng..

[20] Nguyen T-A, Ly H-B (2021). Estimation of the bond strength between FRP and concrete using ANFIS and hybridized ANFIS machine learning models. J. Sci. Transp. Technol..

[21] Kainthura P, Sharma N (2022). Hybrid machine learning approach for landslide prediction, Uttarakhand India. Sci. Rep..

[22] Ahmad J (2021). Effects of waste glass and waste marble on mechanical and durability performance of concrete. Sci. Rep..

[23] Martínez-Álvarez F, Troncoso A, Riquelme JC (2022). Data science and big data in energy forecasting. Energies.

[24] Amini Pishro A (2021). Application of artificial neural networks and multiple linear regression on local bond stress equation of UHPC and reinforcing steel bars. Sci. Rep..

[25] Wakjira TG, Abushanab A, Ebead U, Alnahhal W (2022). FAI: Fast, accurate, and intelligent approach and prediction tool for flexural capacity of FRP-RC beams based on super-learner machine learning model. Mater. Today Commun..

[26] Wakjira TG, Ebead U, Alam MS (2022). Machine learning-based shear capacity prediction and reliability analysis of shear-critical RC beams strengthened with inorganic composites. Case Stud. Constr. Mater..

[27] Uddin MN (2022). Developing machine learning model to estimate the shear capacity for RC beams with stirrups using standard building codes. Innov. Infrastruct. Solut..

[28] Wakjira TG, Ibrahim M, Ebead U, Alam MS (2022). Explainable machine learning model and reliability analysis for flexural capacity prediction of RC beams strengthened in flexure with FRCM. Eng. Struct..

[29] Badra N, Aboul Haggag SY, Deifalla A, Salem NM (2022). Development of machine learning models for reliable prediction of the punching shear strength of FRP-reinforced concrete slabs without shear reinforcements. Measurement.

[30] Deifalla A, Salem NM (2022). A machine learning model for torsion strength of externally bonded FRP-reinforced concrete beams. Polymers.

[31] Mohammed HRM, Ismail S (2022). Proposition of new computer artificial intelligence models for shear strength prediction of reinforced concrete beams. Eng. Comput..

[32] Salem NM, Deifalla A (2022). Evaluation of the strength of slab-column connections with FRPs using machine learning algorithms. Polymers.

[33] Ebid A, Deifalla A (2022). Using artificial intelligence techniques to predict punching shear capacity of lightweight concrete slabs. Materials.

[34] Kaveh, A., Mohammad Javadi, S. & Mahdipour Moghani, R. Shear strength prediction of FRP-reinforced concrete beams using an extreme gradient boosting framework. *Period. Polytech. Civ. Eng.* **66**, 18–29. 10.3311/PPci.18901 (2022).

[35] GB50010-2002. Code for design of concrete structures. *China Construction Industry* (2002).

[36] China Academy of building Research, Design and Construction of Reinforced Concrete Structure: Compilation of Background Data for Design Code-1985, Beijing Sanhuan Printing Plant, 1985 (in Chinese).

[37] Zararis PD (2003). Shear compression failure in reinforced concrete deep beams. J. Struct. Eng..

[38] Lu Z-H, Li H, Li W, Zhao Y-G, Dong W (2018). An empirical model for the shear strength of corroded reinforced concrete beam. Constr. Build. Mater..

[39] Tanabe T, Higai T, Umehara H, Niwa J (2000). Concrete structure.

[40] Futaha, Jun., Yamada, K., Yokozawa, K. & Okamura, H. Re-evaluation of shear strength formula of RC beams without shear reinforcement. *J. Japan Soc. Civ. Eng*. **1**, 372. 10.2208/jscej.1986.372_167 (1986).

[41] Niwa, J. Shear equation of deep beams based on analysis. In *Proceedings of JCI 2nd Colloquium on Shear Analysis of RC Structures, Tokyo* (1983).

[42] Rodriguez J, Ortega LM, Casal J (1997). Load carrying capacity of concrete structures with corroded reinforcement. Constr. Build. Mater..

[43] Higgins C, Farrow WC (2006). Tests of reinforced concrete beams with corrosion-damaged stirrups. ACI Mater. J..

[44] Xia J, Jin W-L, Li L-Y (2011). Shear performance of reinforced concrete beams with corroded stirrups in chloride environment. Corros. Sci..

[45] Imam A, Azad AK (2016). Prediction of residual shear strength of corroded reinforced concrete beams. Int. J. Adv. Struct. Eng..

[46] Juarez CA, Guevara B, Fajardo G, Castro-Borges P (2011). Ultimate and nominal shear strength in reinforced concrete beams deteriorated by corrosion. Eng. Struct..

[47] Liu, S. *The research on shear capacity of corroded rc beams*, PhD Thesis, Master's thesis, Central South University, China, (2013).

[48] Singh R (2022). Enhancing sustainability of corroded RC structures: Estimating steel-to-concrete bond strength with ANN and SVM algorithms. Materials.

[49] Kumar, A., Arora, H. C., Kapoor, N. R. & Kumar, K. Prognosis of compressive strength of fly-ash-based geopolymer-modified sustainable concrete with ML algorithms. *Struct. Concrete.*10.1002/suco.202200344.

[50] Kumar A, Arora HC, Kumar K, Garg H (2023). Performance prognosis of FRCM-to-concrete bond strength using ANFIS-based fuzzy algorithm. Expert Syst. Appl..

[51] Liu Q-F (2021). Prediction of chloride diffusivity in concrete using artificial neural network: Modelling and performance evaluation. Constr. Build. Mater..

[52] Ebid AM, Deifalla AF, Mahdi HA (2022). Evaluating shear strength of light-weight and normal-weight concretes through artificial intelligence. Sustainability.

[53] Kurtgoz, Y. & Deniz, E. in *Exergetic, Energetic and Environmental Dimensions* (eds Ibrahim Dincer, C. Ozgur Colpan, & Onder Kizilkan) 133–148 (Academic Press, 2018).

[54] Amirkhani S, Nasirivatan S, Kasaeian AB, Hajinezhad A (2015). ANN and ANFIS models to predict the performance of solar chimney power plants. Renew. Energy.

[55] Kumar K, Saini RP (2022). Adaptive neuro-fuzzy interface system based performance monitoring technique for hydropower plants. ISH J. Hydraul. Eng..

[56] Buragohain M, Mahanta C (2008). A novel approach for ANFIS modelling based on full factorial design. Appl. Soft Comput..

[57] Zhang J, Li J, Hu Y, Zhou JY (2012). The identification method of igneous rock lithology based on data mining technology. Adv. Mater. Res..

[58] Wakjira TG, Al-Hamrani A, Ebead U, Alnahhal W (2022). Shear capacity prediction of FRP-RC beams using single and ensenble ExPlainable Machine learning models. Compos. Struct..

[59] Chen, T. & Guestrin, C. in *Proceedings of the 22nd acm sigkdd international conference on knowledge discovery and data mining* (pp. 785–794).

[60] Wakjira TG, Alam MS, Ebead U (2021). Plastic hinge length of rectangular RC columns using ensemble machine learning model. Eng. Struct..

[61] Fu B, Feng D-C (2021). A machine learning-based time-dependent shear strength model for corroded reinforced concrete beams. J. Build. Eng..

[62] Chen, T., & Guestrin, C. Xgboost: A scalable tree boosting system. In *Proceedings of the 22nd acm sigkdd international conference on knowledge discovery and data mining* (pp. 785–794). 10.1145/2939672.2939785 (2016).

