# Supplementary material for: Machine learning intelligence to assess the shear capacity of corroded reinforced concrete beams
Source: Sci Rep. 2023 Feb 17;13:2857. doi: 10.1038/s41598-023-30037-9 (PMC9938144; doi:10.1038/s41598-023-30037-9)
Supplement: Supplementary file 1 — Supplementary Information. [file 41598_2023_30037_MOESM1_ESM.docx]

**SUPPLEMENTARY DATA**

**Machine Learning Intelligence to Assess the Shear Capacity of Corroded Reinforced Concrete Beams**

*Aman* ***Kumar****^1,2^, Harish Chandra* ***Arora****^1,2^, Nishant Raj* ***Kapoor****^1,3^, Krishna* ***Kumar****^4^, Marijana* ***Hadzima-Nyarko****^5,6^, and Dorin* ***Radu****^6^*

*^1^Academy of Scientific and Innovative Research (AcSIR) Ghaziabad 201002, India;* [*aman.civil16@outlook.com*](mailto:aman.civil16@outlook.com)

*^2^Structural Engineering Department, CSIR*—*Central Building Research Institute Roorkee 247667, India;* [*hcarora@cbri.res.in*](mailto:hcarora@cbri.res.in)

*^3^Department of Architecture and Planning, CSIR*—*Central Building Research Institute Roorkee 247667, India;* [*dr.nrkapoor@outlook.com*](mailto:dr.nrkapoor@outlook.com)

*^4^Department of Hydro and Renewable Energy Indian Institute of Technology Roorkee, 247667, India;* [*krishnanitald@gmail.com*](mailto:krishnanitald@gmail.com)

*^5^Faculty of Civil Engineering and Architecture Osijek, J. J. Strossmayer University of Osijek, Vladimira Preloga, Croatia;* [*mhadzima@gfos.hr*](mailto:mhadzima@gfos.hr)

*^6^Faculty of Civil Engineering, Transilvania University of Brașov, Romania;* [*dorin.radu@unitbv.ro*](mailto:dorin.radu@unitbv.ro)

**Table 1.** Selection of best neuron

| **Neuron** | **Values** | | | | | | **Rank** | | | | **Total** |
| --- | --- | --- | --- | --- | --- | --- | --- | --- | --- | --- | --- |
|  | **R** | | | **MSE** | | | **R** | | **MSE** | |  |
|  | **Training** | **Testing** | **All** | **Training** | **Testing** | **All** | **Training** | **Testing** | **Training** | **Testing** |  |
| 3 | 0.9751 | 0.7104 | 0.9713 | 0.0039 | 0.0151 | 0.0056 | 8 | 8 | 8 | 9 | 33 |
| 4 | 0.9957 | 0.6777 | 0.9914 | 0.0012 | 0.0039 | 0.0017 | 4 | 9 | 5 | 4 | 22 |
| 5 | 0.9955 | 0.9748 | 0.9914 | 0.0010 | 0.0015 | 0.0017 | 5 | 4 | 4 | 3 | 16 |
| 6 | 0.9973 | 0.9628 | 0.9914 | 0.0006 | 0.0013 | 0.0017 | 2 | 5 | 2 | 2 | 11 |
| 7 | 0.9935 | 0.9384 | 0.9914 | 0.0017 | 0.0046 | 0.0017 | 6 | 7 | 6 | 5 | 24 |
| 8 | 0.9927 | 0.9534 | 0.9914 | 0.0024 | 0.0047 | 0.0017 | 7 | 6 | 7 | 6 | 26 |
| 9 | 0.9739 | 0.9771 | 0.9914 | 0.0043 | 0.0134 | 0.0017 | 9 | 3 | 9 | 8 | 29 |
| 10 | 0.9968 | 0.9945 | 0.9914 | 0.0007 | 0.0011 | 0.0017 | 3 | 1 | 3 | 1 | 8 |
| 11 | 0.9975 | 0.9896 | 0.9914 | 0.0005 | 0.0059 | 0.0017 | 1 | 2 | 1 | 7 | 11 |

**Table 2.** Selection of cluster center

| **Data From ANFIS MODEL** | | | | | | | | | | | | | | | | | **Ranking** | | | | **Total** | **Rank** |
| --- | --- | --- | --- | --- | --- | --- | --- | --- | --- | --- | --- | --- | --- | --- | --- | --- | --- | --- | --- | --- | --- | --- |
| **S. No.** | **r** | **SF** | **R_u_** | **RMSE (nor.)** | **R** | | | **MAPE** | | | **RMSE** | | | **MAE** | | | **R** | **MAPE** | **RMSE** | **MAE** |  |  |
|  |  |  |  |  | **Tr.** | **Te.** | **All** | **Tr.** | **Te.** | **All** | **Tr.** | **Te.** | **All** | **Tr.** | **Te.** | **All** |  |  |  |  |  |  |
| 1 | 0.9 | 1.25 | 9 | 0.01674 | 0.9979 | 0.8835 | 0.9369 | 3.3185 | 14.0906 | 6.5502 | 4.7507 | 57.3033 | 31.6370 | 3.2741 | 20.0480 | 8.3063 | 13 | 10 | 13 | 13 | 49 | 13 |
| 2 | 0.85 | 1.25 | 10 | 0.01648 | 0.9971 | 0.9557 | 0.9702 | 3.2038 | 13.1443 | 6.1859 | 4.6767 | 41.1576 | 22.8800 | 3.1660 | 17.0319 | 7.3258 | 11 | 7 | 10 | 10 | 38 | 10 |
| 3 | 0.8 | 1.25 | 10 | 0.01177 | 0.9989 | 0.9559 | 0.9758 | 2.1826 | 15.9724 | 6.3196 | 3.3398 | 35.8989 | 19.8602 | 1.8574 | 18.6412 | 6.8925 | 8 | 8 | 8 | 9 | 33 | 7 |
| 4 | 0.75 | 1.25 | 10 | 0.01171 | 0.9989 | 0.9521 | 0.9738 | 2.1574 | 17.4739 | 6.7523 | 3.3239 | 37.2868 | 20.6113 | 1.8339 | 20.4698 | 7.4246 | 9 | 11 | 9 | 11 | 40 | 11 |
| 5 | 0.7 | 1.25 | 11 | 0.01169 | 0.9989 | 0.9597 | 0.9711 | 2.1439 | 15.3512 | 6.1061 | 3.3185 | 45.0758 | 24.8447 | 1.8309 | 17.8714 | 6.6431 | 10 | 5 | 11 | 8 | 34 | 8 |
| 6 | 0.65 | 1.25 | 12 | 0.01172 | 0.9989 | 0.9502 | 0.9614 | 2.1663 | 15.4088 | 6.1391 | 3.3266 | 48.0229 | 26.4501 | 1.8572 | 21.3671 | 7.7101 | 12 | 6 | 12 | 12 | 42 | 12 |
| 7 | 0.6 | 1.25 | 13 | 0.01416 | 0.9985 | 0.4781 | 0.7160 | 2.8316 | 40.0562 | 13.9990 | 4.0189 | 125.3227 | 68.7244 | 2.5710 | 53.8646 | 17.9591 | 15 | 15 | 15 | 15 | 60 | 15 |
| 8 | 0.55 | 1.25 | 16 | 0.01360 | 0.9986 | 0.7818 | 0.8325 | 2.6053 | 17.7332 | 7.1437 | 3.8604 | 91.4469 | 50.1916 | 2.2796 | 33.0288 | 11.5043 | 14 | 13 | 14 | 14 | 55 | 14 |
| 9 | 0.5 | 1.25 | 18 | 0.01211 | 0.9989 | 0.9874 | 0.9928 | 2.0913 | 13.5527 | 5.5297 | 3.4367 | 18.8972 | 10.7424 | 1.7910 | 12.6807 | 5.0579 | 2 | 3 | 1 | 2 | 8 | 2 |
| 10 | 0.45 | 1.25 | 18 | 0.01309 | 0.9987 | 0.9894 | 0.9933 | 2.3935 | 12.6229 | 5.4623 | 3.7146 | 18.8091 | 10.7608 | 2.0639 | 11.7552 | 4.9713 | 1 | 1 | 2 | 1 | 5 | 1 |
| 11 | 0.4 | 1.25 | 23 | 0.013206 | 0.9987 | 0.9772 | 0.9874 | 2.4023 | 16.9242 | 6.7589 | 3.7466 | 25.4505 | 14.2879 | 2.0829 | 15.9877 | 6.2543 | 6 | 12 | 6 | 6 | 30 | 6 |
| 12 | 0.35 | 1.25 | 31 | 0.013198 | 0.9987 | 0.9811 | 0.9886 | 2.3978 | 15.5318 | 6.3380 | 3.7442 | 24.8425 | 13.9628 | 2.0768 | 15.0956 | 5.9825 | 5 | 9 | 5 | 5 | 24 | 5 |
| 13 | 0.3 | 1.25 | 38 | 0.011589 | 0.9981 | 0.9846 | 0.9902 | 1.8182 | 15.6488 | 5.9674 | 3.2879 | 23.6103 | 13.2212 | 1.7294 | 14.7069 | 5.6227 | 4 | 4 | 4 | 4 | 16 | 4 |
| 14 | 0.25 | 1.25 | 48 | 0.011479 | 0.9991 | 0.9884 | 0.9919 | 1.7748 | 14.2381 | 5.5138 | 3.2566 | 21.8267 | 12.2615 | 1.6806 | 13.2917 | 5.1639 | 3 | 2 | 3 | 3 | 11 | 3 |
| 15 | 0.2 | 1.25 | 52 | 0.01065 | 0.9991 | 0.9685 | 0.9825 | 1.5151 | 20.6396 | 7.2525 | 3.0213 | 30.4131 | 16.8486 | 1.3800 | 17.6369 | 6.2571 | 7 | 14 | 7 | 7 | 35 | 9 |

R_u_ = no. of rules; nor. =normalized, Tr. = Training; Te. = Testing

**Figures Extracted from ANFIS Model**

**Fig. 1.** 3-D interaction diagram between *V_u_* (output) and two input parameters (one input = *b*);- **(a)** *b* and *d*; **(b)** *b* and f_ck_ ; **(c)** *b* and *fy* ; **(d)** *b* and $\rho_{l}$ **(e)** *b* and $\rho_{v}$ **(f)** *b* and $f_{yv}$ **(g)** *b* and *s*; **(h)** *b* and $\lambda$ **(i)** *b* and $\eta_{l}$; and **(j)** *b* and $\eta_{w}$

**Fig. 2.** 3-D interaction diagram between *V_u_* (output) and two input parameters (one input = *d*);- **(a)** *d* and f_ck_ ; **(b)** *d* and *fy* ; **(c)** *d* and $\rho_{l}$ **(d)** *d* and $\rho_{v}$ **(e)** *d* and $f_{yv}$ **(f)** *d* and *s*; **(g)** *d* and $\lambda$ **(h)** *d* and $\eta_{l}$; and **(i)** *d* and $\eta_{w}$

**Fig. 3.** 3-D interaction diagram between *V_u_* (output) and two input parameters (one input = *f_ck_*);- **(a)** *f_ck_* and *fy* ; **(b)** *f_ck_* and $\rho_{l}$ **(c)** *f_ck_* and $\rho_{v}$ **(d)** *f_ck_* and $f_{yv}$ **(e)** *f_ck_* and *s*; **(f)** *f_ck_* and $\lambda$ **(g)** *f_ck_* and $\eta_{l}$; and **(h)** *f_ck_* and $\eta_{w}$

**Fig. 4.** 3-D interaction diagram between *V_u_* (output) and two input parameters (one input = *f_y_*);- **(a)** *f_y_* and $\rho_{l}$ **(b)** *f_y_* and $\rho_{v}$ **(c)** *f_y_* and $f_{yv}$ **(d)** *f_y_* and *s*; **(e)** *f_y_* and $\lambda$ **(f)** *f_y_* and $\eta_{l}$; and **(g)** *f_y_* and $\eta_{w}$

**Fig. 5.** 3-D interaction diagram between *V_u_* (output) and two input parameters;- **(a)** $\rho_{l}$ and $\rho_{v}$ **(b)** $\rho_{l}$ and $f_{yv}$ **(c)** $\rho_{l}$ and *s*; **(d)** $\rho_{l}$and $\lambda$ **(e)** $\rho_{l}$ and $\eta_{l}$; **(f)** $\rho_{l}$ and $\eta_{w}$; **(g)** $\rho_{v}$ and $f_{yv}$ **(h)** $\rho_{v}$ and *s*; **(i)** $\rho_{v}$and $\lambda$ **(j)** $\rho_{v}$ and $\eta_{l}$; and **(k)** $\rho_{v}$ and $\eta_{w}$

**Fig. 6.** 3-D interaction diagram between *V_u_* (output) and two input parameters;- **(a)** $f_{yv}$ and *s*; **(b)** $f_{yv}$and $\lambda$ **(c)** $f_{yv}$ and $\eta_{l}$; **(d)** $f_{yv}$ and $\eta_{w}$; **(e)** $s$and $\lambda$ **(f)** *s* and $\eta_{l}$; **(g)** $s$ and $\eta_{w}$; **(h)** $\lambda$ and $\eta_{l}$; **(i)** $\lambda$ and $\eta_{w}$; and **(j)** $\eta_{l}$ and $\eta_{w}$

**Fig. 1.** 3-D interaction diagram between *V_u_* (output) and two input parameters (one input = *b*);- **(a)** *b* and *d*; **(b)** *b* and f_ck_ ; **(c)** *b* and *fy* ; **(d)** *b* and $\rho_{l}$ **(e)** *b* and $\rho_{v}$ **(f)** *b* and $f_{yv}$ **(g)** *b* and *s*; **(h)** *b* and $\lambda$ **(i)** *b* and $\eta_{l}$; and **(j)** *b* and $\eta_{w}$

**Fig. 2.** 3-D interaction diagram between *V_u_* (output) and two input parameters (one input = *d*);- **(a)** *d* and f_ck_ ; **(b)** *d* and *fy* ; **(c)** *d* and $\rho_{l}$ **(d)** *d* and $\rho_{v}$ **(e)** *d* and $f_{yv}$ **(f)** *d* and *s*; **(g)** *d* and $\lambda$ **(h)** *d* and $\eta_{l}$; and **(i)** *d* and $\eta_{w}$

**Fig. 3.** 3-D interaction diagram between *V_u_* (output) and two input parameters (one input = *f_ck_*);- **(a)** *f_ck_* and *fy* ; **(b)** *f_ck_* and $\rho_{l}$ **(c)** *f_ck_* and $\rho_{v}$ **(d)** *f_ck_* and $f_{yv}$ **(e)** *f_ck_* and *s*; **(f)** *f_ck_* and $\lambda$ **(g)** *f_ck_* and $\eta_{l}$; and **(h)** *f_ck_* and $\eta_{w}$

**Fig. 4.** 3-D interaction diagram between *V_u_* (output) and two input parameters (one input = *f_y_*);- **(a)** *f_y_* and $\rho_{l}$ **(b)** *f_y_* and $\rho_{v}$ **(c)** *f_y_* and $f_{yv}$ **(d)** *f_y_* and *s*; **(e)** *f_y_* and $\lambda$ **(f)** *f_y_* and $\eta_{l}$; and **(g)** *f_y_* and $\eta_{w}$

**Fig. 5.** 3-D interaction diagram between *V_u_* (output) and two input parameters;- **(a)** $\rho_{l}$ and $\rho_{v}$ **(b)** $\rho_{l}$ and $f_{yv}$ **(c)** $\rho_{l}$ and *s*; **(d)** $\rho_{l}$and $\lambda$ **(e)** $\rho_{l}$ and $\eta_{l}$; **(f)** $\rho_{l}$ and $\eta_{w}$; **(g)** $\rho_{v}$ and $f_{yv}$ **(h)** $\rho_{v}$ and *s*; **(i)** $\rho_{v}$and $\lambda$ **(j)** $\rho_{v}$ and $\eta_{l}$; and **(k)** $\rho_{v}$ and $\eta_{w}$

**Fig. 6.** 3-D interaction diagram between *V_u_* (output) and two input parameters;- **(a)** $f_{yv}$ and *s*; **(b)** $f_{yv}$and $\lambda$ **(c)** $f_{yv}$ and $\eta_{l}$; **(d)** $f_{yv}$ and $\eta_{w}$; **(e)** $s$and $\lambda$ **(f)** *s* and $\eta_{l}$; **(g)** $s$ and $\eta_{w}$; **(h)** $\lambda$ and $\eta_{l}$; **(i)** $\lambda$ and $\eta_{w}$; and **(j)** $\eta_{l}$ and $\eta_{w}$
